# Comprehensive Tools for Culturing *Blastocystis*: A Standardized Resource for Research and Diagnostics

**DOI:** 10.1002/cpz1.70175

**Published:** 2025-08-14

**Authors:** Daisy Shaw, Constance Denoyelle, Kevin S. W. Tan, C. Graham Clark, Hisao Yoshikawa, Eric Viscogliosi, Eleni Gentekaki, Kateřina Jirků, Anastasios D. Tsaousis

**Affiliations:** ^1^ Laboratory of Molecular and Evolutionary Parasitology, School of Natural Sciences University of Kent Canterbury Kent United Kingdom; ^2^ Univ. Lille, CNRS, Inserm, CHU Lille, Institut Pasteur de Lille, U1019–UMR 9017–Centre d'Infection et d'Immunité de Lille (CIIL) Lille France; ^3^ Department of Microbiology and Immunology, Yong Loo Lin School of Medicine National University of Singapore Singapore Singapore; ^4^ Faculty of Infectious and Tropical Diseases London School of Hygiene and Tropical Medicine London United Kingdom; ^5^ Department of Global Infectious Diseases, Graduate School of Medical Sciences Kanazawa University Kanazawa Japan; ^6^ Department of Veterinary Medicine University of Nicosia School of Veterinary Medicine Nicosia Cyprus; ^7^ Institute of Parasitology Biology Centre of the Czech Academy of Sciences (BC CAS) České Budějovice Czech Republic

**Keywords:** blastocystis, in vitro, culturing

## Abstract

*Blastocystis* spp. is a widely prevalent anaerobic protozoan of uncertain pathogenicity found in the gastrointestinal tracts of over 1 billion people worldwide. Despite its potential significance in health and disease, *Blastocystis* spp. remains challenging to culture axenically due to its anaerobic nature and the diversity of its genetic subtypes. This manuscript presents a standardized toolkit for culturing *Blastocystis* spp. in xenic and axenic conditions, detailing protocols for the preparation of appropriate liquid and solid media, cryopreservation, and inoculation. By providing a comprehensive set of tools and methodologies, this work aims to streamline research on *Blastocystis* spp., enabling reproducibility, subtype characterization, and advancements in understanding its role in the gut microbiome and host health. © 2025 The Author(s). Current Protocols published by Wiley Periodicals LLC.

**Basic Protocol 1**: Establishment of xenic *Blastocystis* culture from stool samples in liquid medium

**Basic Protocol 2**: Subculturing from existing *Blastocystis* liquid culture

**Support Protocol 1**: Preparation of modified Jones’ medium for *Blastocystis* culturing

**Support Protocol 2**: Preparation of trypticase‐yeast extract‐serum‐gastric mucin‐9 (TSGYM‐9) medium for *Blastocystis* culturing

**Support Protocol 3**: Preparation of liver extract‐yeast extract‐serum‐gastric mucin (LYSGM) medium for *Blastocystis* culturing

**Basic Protocol 3**: Subculturing xenic *Blastocystis* cultures in diphasic medium

**Support Protocol 4**: Preparation of Robinson's medium for *Blastocystis* culturing

**Support Protocol 5**: Preparation of Ringer's agar slants for *Blastocystis* culturing

**Basic Protocol 4**: Axenization of *Blastocystis* cultures

**Basic Protocol 5**: Subculturing axenic *Blastocystis* cultures in diphasic medium

**Support Protocol 6**: Preparation of Boeck and Drbohlav's Locke‐egg serum (LES) medium for *Blastocystis* culturing

**Support Protocol 7**: Preparation of Boeck and Drbohlav's diphasic modified medium (BDMM) for *Blastocystis* culturing

**Basic Protocol 6**: Establishment of axenic *Blastocystis* cultures in Iscove's modified Dulbecco's medium (IMDM)

**Basic Protocol 7**: Establishment of axenic *Blastocystis* cultures in soft IMDM agar

**Basic Protocol 8**: Establishment of axenic *Blastocystis* cultures on solid IMDM agar

**Basic Protocol 9**: Optimized method for establishing axenic *Blastocystis* cultures on solid IMDM agar

**Basic Protocol 10**: Establishment of axenic *Blastocystis* cultures in semi‐solid Locke's agar

**Basic Protocol 11**: Cryopreservation of xenic *Blastocystis* cultures

**Basic Protocol 12**: Cryopreservation of axenic *Blastocystis* cultures

**Basic Protocol 13**: Inoculation of liquid medium with xenic *Blastocystis* cultures from frozen stocks

**Basic Protocol 14**: Inoculation of liquid medium with axenic *Blastocystis* cultures from frozen stocks

## Introduction

*Blastocystis* spp. is an anaerobic gastrointestinal protozoan of considerable interest due to its widespread prevalence and controversial role in health and disease. It is estimated to inhabit the guts of over 1 billion individuals globally (Scanlan & Stensvold, 2013), spanning both asymptomatic carriers and individuals with gastrointestinal disturbances, such as diarrhea and abdominal pain. The duality of its presence in healthy and symptomatic individuals continues to fuel the debate about whether *Blastocystis* spp. acts as a commensal organism or a pathogen.

The diversity of its subtypes (STs) further underscores the complexity of *Blastocystis* spp. To date, at least 44 STs have been identified in mammalian and avian hosts (Šejnohová et al., 2024), based on gene polymorphisms in the small subunit ribosomal RNA gene (*SSU* rDNA; Scicluna et al., 2006), with ST1‐ST4 being most commonly found in humans. This heterogeneity highlights the importance of ST‐specific studies, particularly in understanding transmission pathways, ecological niches, and host‐specific interactions.

Culturing *Blastocystis* spp. for research purposes has historically been challenging because of its anaerobic growth requirements and dependence on coexisting microorganisms for xenic cultivation. Although a limited number of STs have been axenized successfully, maintaining viable cultures often necessitates xenic conditions, in which other microbes play a critical role in oxygen consumption and nutrient provision. Overcoming these barriers with standardized and reproducible culturing techniques is essential for advancing research into *Blastocystis* spp. biology, its interactions with the gut microbiome, and its potential impact on host health.

**Figure 1:**
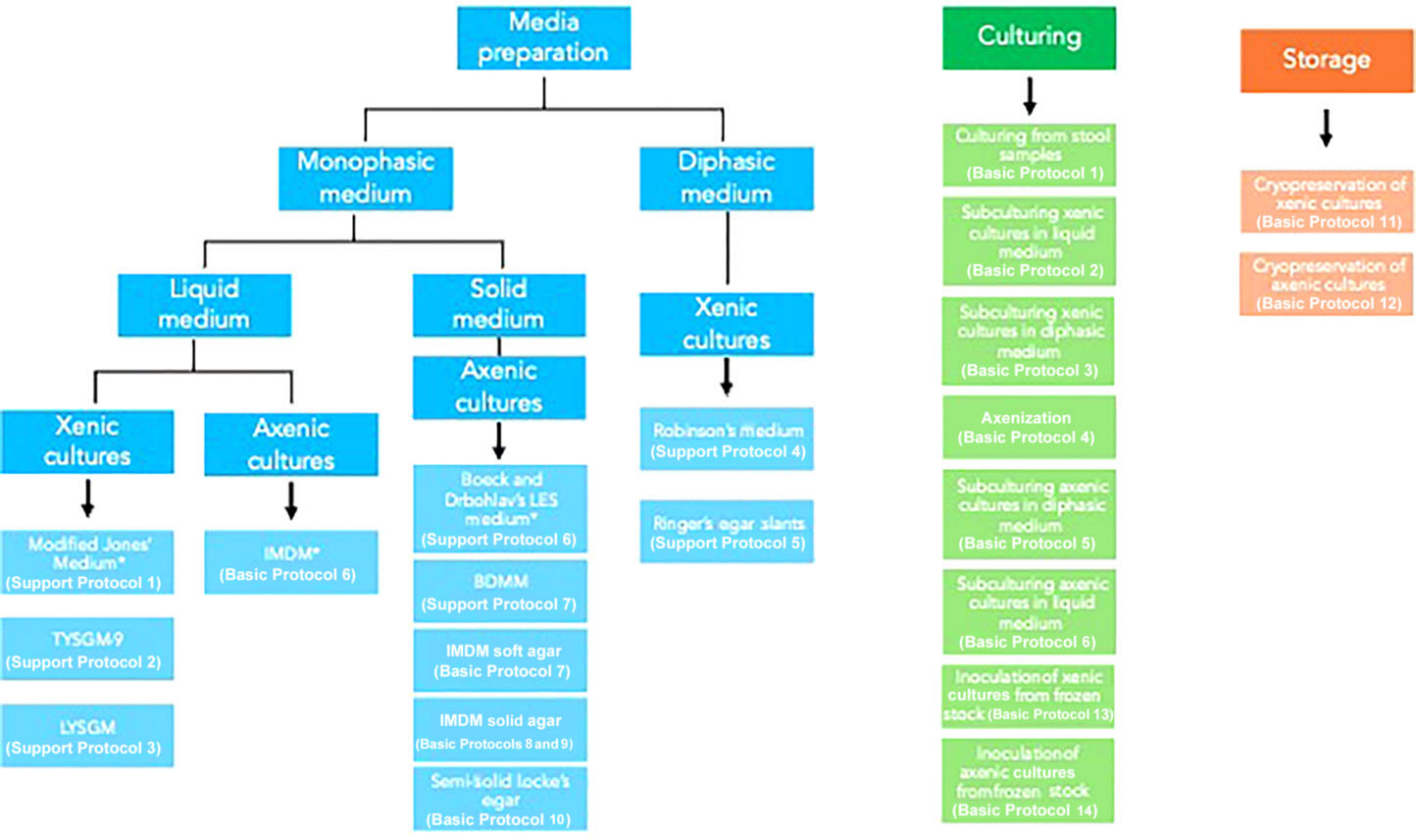
Schematic of the topics covered in this paper.

This article consolidates the current knowledge of and methods for culturing *Blastocystis* spp., offering a comprehensive guide to both xenic and axenic cultivation techniques (definitions of some key terms used in this article are provided in Table 1). By detailing protocols for the preparation of liquid and solid media, growth monitoring, and cryopreservation (see Fig. 1), we aim to equip the scientific community with the tools necessary for consistent and successful initiation and maintenance of *Blastocystis* spp. cultures. The article consists of eight main parts: medium preparation and methods for xenic cultures in liquid media (Basic Protocols 1 and 2 and Support Protocols 1, 2, and 3); medium preparation and methods for xenic cultures in diphasic media (Basic Protocol 3 and Support Protocols 4 and 5); axenization (Basic Protocol 4); medium preparation and methods for axenic cultures in diphasic media (Basic Protocol 5 and Support Protocols 6 and 7); medium preparation and culturing methods for axenic cultures in liquid media (Basic Protocol 6); medium preparation and methods for axenic cultures in semi‐solid and solid media (Basic Protocols 7‐9); cryopreservation (Basic Protocols 10 and 11); and establishment of xenic and axenic cultures from frozen stocks (Basic Protocols 12 and 13). This work addresses the current bottlenecks in *Blastocystis* research and facilitates further exploration into its enigmatic role in the gut microbiome.

**Table 1:** Key Terms

Anaerobic	An environment that is completely free of oxygen
Axenic	A culture of *Blastocystis* devoid of other microbial contaminants (bacteria, fungi, etc.)
Cryopreservation	The process of preserving biological samples at extremely low temperatures to maintain viability over time
Diphasic	A type of culturing system that includes both a solid and liquid phase
Inspissation	A technique involving the coagulation of medium via heating
Microaerophilic	Conditions with very low levels of oxygen, but not fully anaerobic
Monophasic	A type of culturing system consisting of a single phase (solid, semi‐solid, or liquid)
Pre‐reducing	The process of removing oxygen from culture medium before inoculation, typically by storing in an anaerobic chamber
Pre‐warming	Bringing culture medium to incubation temperature before inoculation, preventing thermal shock
Xenic	A culture of *Blastocystis* that contains other microorganisms (bacteria, fungi, etc.) obtained from the original sample

The methods described herein are recommended as a guide. It is important to be aware that different STs might have distinct culture requirements; thus, although these protocols should work for most STs, there may be STs, or strains within STs, that require a certain level of trial and error to obtain an optimized protocol. This is especially true for axenic cultures. This protocol overview does not include discussion of how to interpret results because it is a review of the methods and protocols used within the field; for specific results related to each protocol, please refer to the studies cited therein. This article is designed to provide a comprehensive resource for *Blastocystis* culturing methods that can be implemented throughout laboratories within the field.

## MEDIA AND CULTURING METHODS FOR XENIC CULTURES IN LIQUID MEDIA

### Basic Protocol 1 Establishment of Xenic *Blastocystis* Cultures from Stool Samples in Liquid Medium

**Figure 2:**
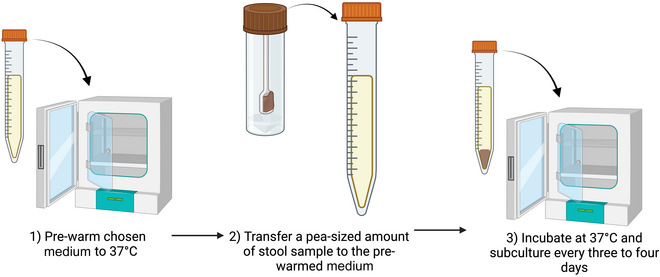
Inoculation of liquid medium from stool samples. Created in BioRender (D. Shaw, 2025; https://BioRender.com/h58m072).

The following procedure should preferably be carried out in a class II biological safety cabinet. See Figure 2 for an overview of the protocol.

#### Materials

Liquid medium
Fresh human stool samples (within 2 hr of collection, or within 24 hr if refrigerated)

Class II biosafety cabinet
Incubator, 37°C

**Figure 3:**
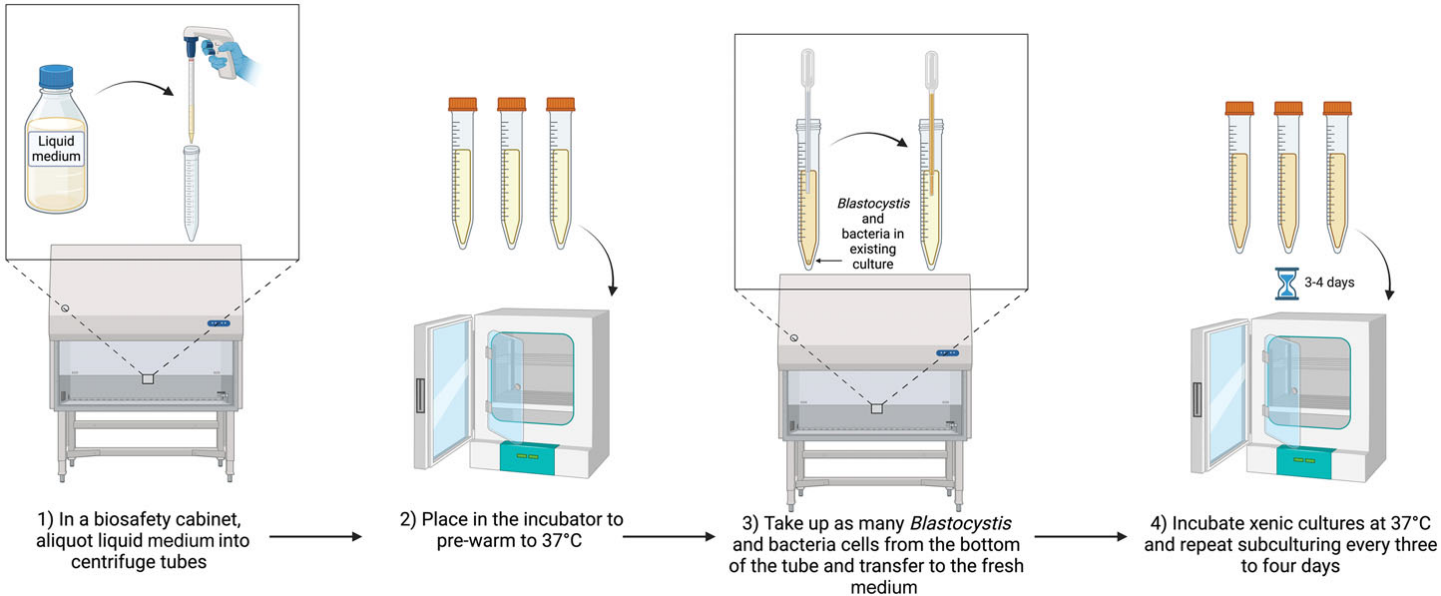
Subculturing of liquid medium from existing liquid cultures. Created in BioRender (D. Shaw, 2025; https://BioRender.com/y69j358).

1 Aliquot 12 ml of your chosen liquid medium into 15‐ml centrifuge tubes and place in a 37°C incubator to pre‐warm.
2 Working in a class II biosafety cabinet, transfer 50‐500 mg (a pea‐sized amount) of fresh stool sample from the collection tube to the pre‐warmed liquid medium.
3 Incubate at 37°C and subculture every 3‐4 days.
  Growth can be variable depending on ST, so frequent observation of the culture under a light microscope should be carried out.
  Results and validation from this protocol can be found in Clark & Stensvold (2016), as well as in Figure 3.

### Basic Protocol 2 Subculturing from Existing *Blastocystis* Liquid Culture

The following procedure should preferably be carried out in a class II biological safety cabinet.

#### Materials

Liquid medium
*Blastocystis* liquid culture

Class II biosafety cabinet
15‐ml centrifuge
Incubator, 37°C

1 Aliquot 12 ml of your chosen liquid medium into 15‐ml centrifuge tubes and place in a 37°C incubator to pre‐warm.
  In the existing culture, *Blastocystis* spp. cells will fall to the bottom of the tube
2 Working in a class II biosafety cabinet, use a sterile transfer pipet to take up *Blastocystis* spp. and bacterial cells from the bottom of the tube.
  The amount transferred can be adjusted depending on cell density.
3 Transfer to the fresh, pre‐warmed medium, avoiding introducing air into the culture.
4 Incubate xenic cultures at 37°C and repeat the subculturing process every 3‐4 days.
  Growth can be variable depending on ST, so frequent observation of the culture under a light microscope should be carried out.
  Results and validation from this protocol can be found in Jones (1946).

### Support Protocol 1 Preparation of Modified Jones’ Medium for *Blastocystis* Culturing

**Figure 4:**
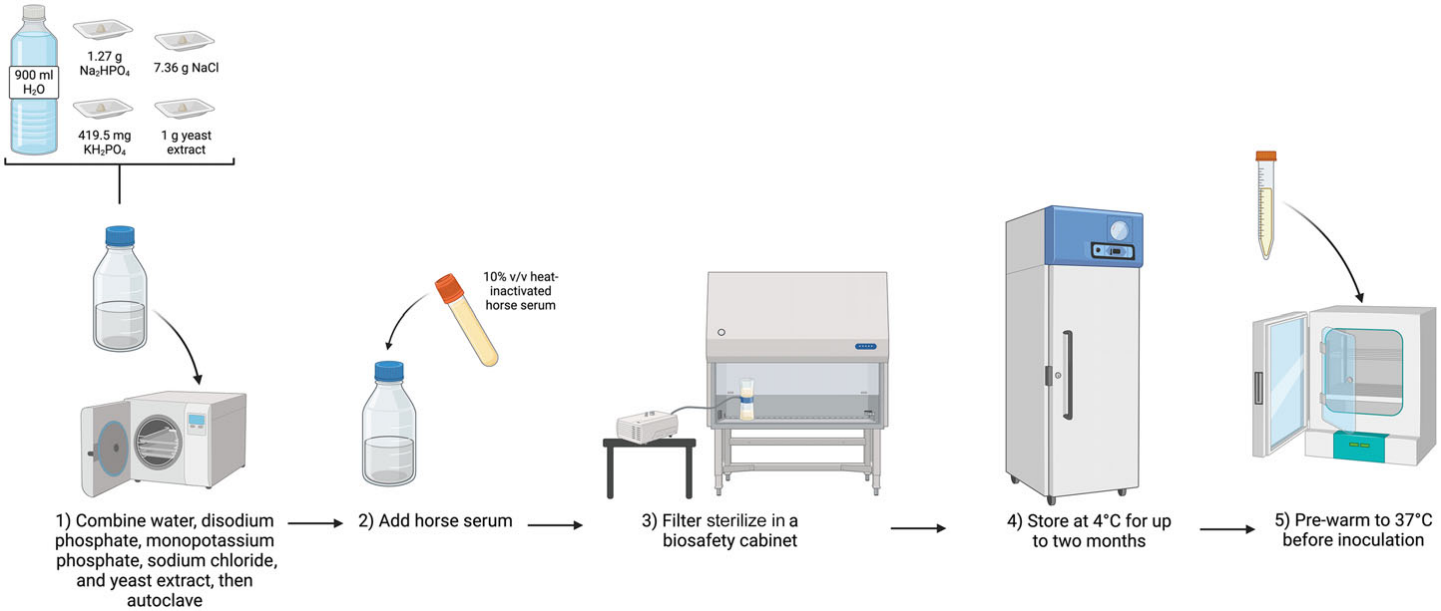
Preparation of modified Jones’ medium for liquid culture. Created in BioRender (D. Shaw, 2025; https://BioRender.com/f80s670).

Xenic cultures of *Blastocystis* spp. can be maintained in a range of different media. The most common monophasic liquid medium is modified Jones’ medium (see Fig. 4), which is based on the study published by W. R. Jones for the cultivation of *Entamoeba histolytica* in 1946 (Jones, 1946). In fact, most of the xenic culturing media used for *Entamoeba* spp. are appropriate for the cultivation of *Blastocystis* spp. (Clark & Diamond, 2002; Leelayoova et al., 2002).

Modified Jones’ medium consists of a buffered saline solution of disodium phosphate, monopotassium phosphate, and sodium chloride mixed with yeast extract and heat‐inactivated horse serum. It is used to cultivate and detect *Blastocystis* spp. from human and animal stool samples, with the organisms’ presence being confirmed by conventional PCR (Lhotská et al., 2020; Šloufová et al., 2022) and qPCR (Šloufová et al., 2022).

***Pros and cons***

Modified Jones’ medium is the standard medium of choice for the maintenance of xenic cultures and is selective for *Blastocystis* over bacteria. It was developed to be used with human samples and is not always optimal for the establishment of cultures from animal samples.

#### Materials

Milli‐Q‐purified water
Disodium phosphate (Na_2_HPO_4_)
Monopotassium phosphate (KH_2_PO_4_)
Sodium chloride (NaCl)
Yeast extract
Horse serum, heat inactivated

1 To prepare modified Jones’ medium, first make the following base solution:
  900 ml Milli‐Q water
  1.27 g Na_2_HPO_4_
  419.5 mg KH_2_PO_4_
  7.36 g NaCl
  1 g yeast extract
2 Autoclave 30 min at 121°C at 15 psi, allow to cool, and add 100 ml heat‐inactivated horse serum to 1 L final volume.
*OPTIONAL*: Base medium can be frozen at –20°C indefinitely, with 10% (v/v) heat‐inactivated horse serum being added before use.
  Many laboratories buy horse serum in non‐heat‐inactivated form; therefore, horse serum should be heat inactivated before being added to any culture medium. Heat inactivation is achieved by heating horse serum for 30 min at 56°C. The serum can then be stored for up to 3 weeks at 4°C or several months at –20°C.
*OPTIONAL*: Working in a sterile environment, filter the medium using a 0.22‐µm‐pore‐size filter.
3 Store medium at 4°C and pre‐warm to 37°C before inoculation with *Blastocystis* spp.
  Results and validation from this protocol can be found in Clark & Diamond (2002), Jones (1946), Leelayoova et al. (2002), Lhotská et al. (2020), and Šloufová et al. (2022).

### Support Protocol 2 Preparation of Trypticase‐Yeast Extract‐Serum‐Gastric Mucin‐9 Medium for *Blastocystis* Culturing

**Figure 5:**
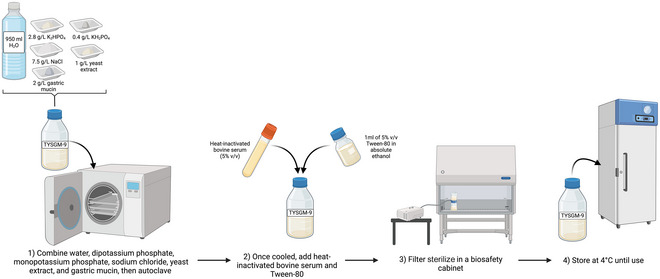
Preparation of TYSM‐9 for liquid culture. Created in BioRender (D. Shaw, 2025; https://BioRender.com/y59e408).

Trypticase‐yeast extract‐serum gastric mucin‐9 (TYSGM‐9) medium (Clark & Diamond, 2002) was derived from trypticase‐yeast extract‐iron‐serum (TYI‐S‐33) medium from Diamond et al. (Diamond, 1982; Gillin & Diamond, 1978), originally developed for axenic culture of *Entamoeba histolytica*, but it is now used for the growth of xenic cultures of *Blastocystis* spp. TYSGM‐9 consists of dipotassium phosphate, monopotassium phosphate, sodium chloride, yeast extract, gastric mucin, heat‐inactivated adult bovine serum, and Tween‐80 (see Fig. 5).

***Pros and cons***

TYSGM‐9 can support both human and animal xenic isolates of *Blastocystis* spp. well, but may not support axenic growth.

#### Materials

Milli‐Q‐purified water
Monopotassium phosphate (K_2_HPO_4_)
Dipotassium phosphate (KH_2_PO_4_)
Sodium chloride (NaCl)
Yeast extract
Gastric mucin
Adult bovine serum, heat inactivated
Tween‐80
Absolute (100%) ethanol

1 To prepare 1 L of TYSGM‐9, combine the following reagents:
  950 ml Milli‐Q water
  2.8 g K_2_HPO_4_
  0.4 g KH_2_PO_4_
  7.5 g NaCl
  1 g yeast extract
  2 g gastric mucin
2 Autoclave 30 min at 121°C, 15 psi, allow to cool, and add 50 ml heat‐inactivated adult bovine serum and 1 ml of 5% (v/v) Tween‐80 in 100% ethanol
*OPTIONAL*: Base medium can be frozen indefinitely at –20°C, with 5% (v/v) heat‐inactivated adult bovine serum being added prior to use.
*OPTIONAL*: Filter the medium in a sterile environment using a 0.22‐µm‐pore‐size filter.
3 Store medium at 4°C and pre‐warm to 37°C before inoculation with *Blastocystis* spp.
  Results and validation from this protocol can be found in Clark & Diamond (2002), Diamond (1982), and Gillin & Diamond (1978).

### Support Protocol 3 Preparation of Liver Extract‐Yeast Extract‐Serum‐Gastric Mucin Medium for *Blastocystis* Culturing

**Figure 6:**
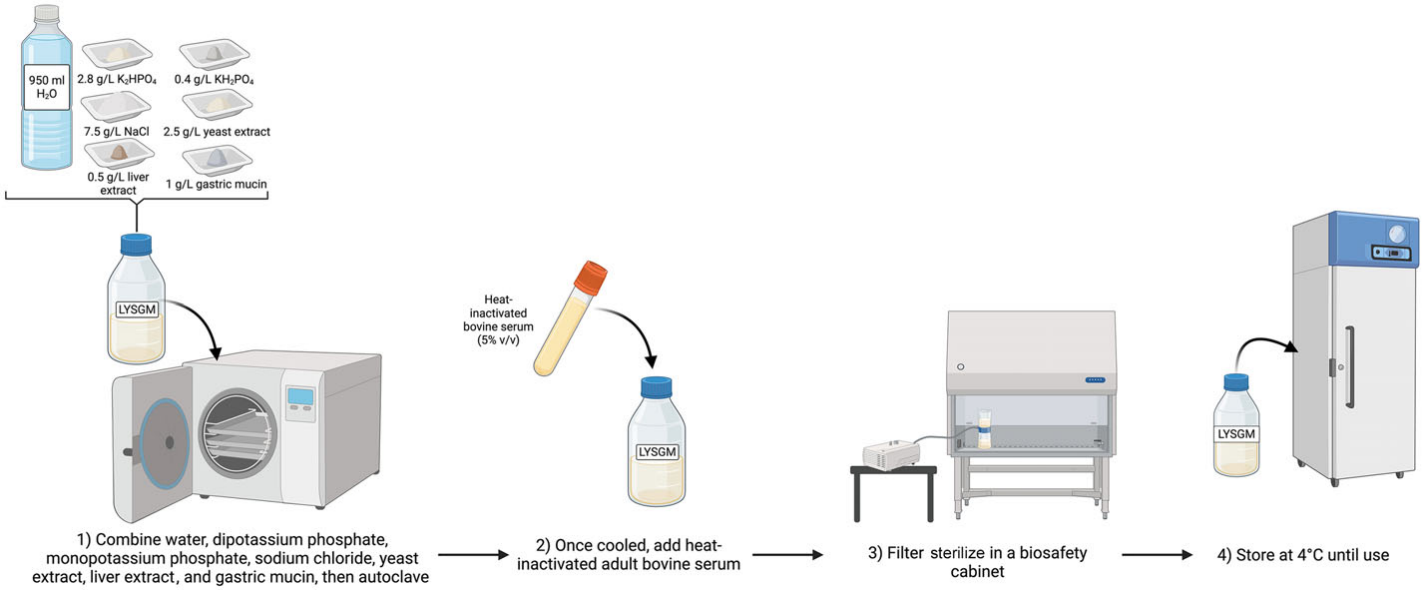
Preparation of LYSGM for liquid culture. Created in BioRender (D. Shaw, 2025; https://BioRender.com/n43a396).

Liver extract‐yeast extract‐serum gastric mucin (LYSGM) medium was derived from trypticase‐yeast extract‐serum‐gastric mucin (TYSGM‐9) medium, which was developed by Diamond et al. for the xenic cultivation of *Entamoeba histolytica* (Diamond, 1982); however, LYSGM medium can support the growth of high densities of *Blastocystis* spp. (Clark & Stensvold, 2016). LYSGM consists of dipotassium phosphate, monopotassium phosphate, sodium chloride, yeast extract, liver extract, gastric mucin, and heat‐inactivated horse serum (see Fig. 6).

***Pros and cons***

LYSGM is rich in nutrients and can support xenic cultures, being especially good for initial isolation from fecal samples; however, its inclusion of complex components such as liver extract, yeast, and serum can lead to batch‐to‐batch variability, so it is recommended that the same lot number be used.

#### Materials

Milli‐Q‐purified water
Monopotassium phosphate (K_2_HPO_4_)
Dipotassium phosphate (KH_2_PO_4_)
Sodium chloride (NaCl)
Yeast extract
Liver extract
Gastric mucin
Adult bovine serum, heat inactivated

1 To prepare 1 L LYSGM, combine the following reagents:
  950 ml Milli‐Q water
  2.8 g K_2_HPO_4_
  0.4 g KH_2_PO_4_
  7.5 g NaCl
  2.5 g yeast extract
  0.5 g liver extract
  1 g gastric mucin
2 Autoclave 30 min at 121°C, 15 psi, allow to cool, and add 50 ml heat‐inactivated adult bovine serum for a final volume of 1 L.
*OPTIONAL*: Base medium can be frozen indefinitely at –20°C, with 5% (v/v) heat‐inactivated adult bovine serum being added before use.
*OPTIONAL*: Filter the medium in a sterile environment using a 0.22‐µm‐pore‐size filter.
3 Store medium at 4°C and pre‐warm to 37°C before inoculation with *Blastocystis* spp.
  Results and validation from this protocol can be found in Clark & Stensvold (2016) and Diamond (1982).

## MEDIA AND CULTURING METHODS FOR XENIC CULTURES IN DIPHASIC MEDIA

Xenic cultures of *Blastocystis* spp. can also be maintained in diphasic media, which consist of two media with distinct physical states: i.e., a solid component topped with a liquid component.

Examples of diphasic medium used for *Blastocystis* spp. include Robinson's medium (Support Protocol 4; Clark & Stensvold, 2016; Robinson, 1968) and Ringer's agar slants (Support Protocol 5; Stenzel & Boreham, 1996).

### Basic Protocol 3 Subculturing Xenic *Blastocystis* Cultures in Diphasic Media

**Figure 7:**
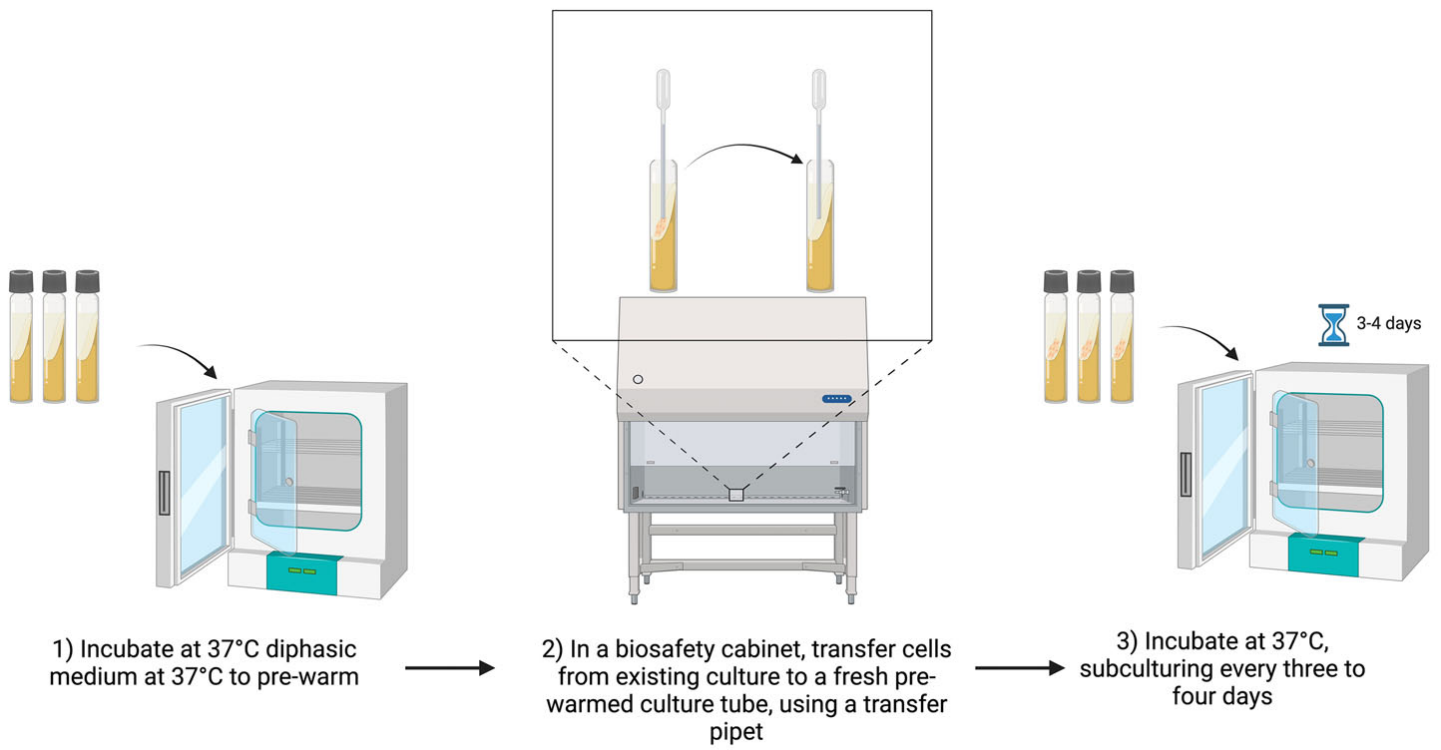
Culturing of xenic STs in diphasic medium. Created in BioRender (D. Shaw, 2025; https://BioRender.com/x93j637).

This procedure is based on that of Robinson (1968). It should preferably be carried out in a class II biological safety cabinet (Fig. 7).

#### Materials

*Blastocystis* spp. culture in diphasic medium
Fresh diphasic medium in fresh culture tubes
Class II biosafety cabinet
Incubator, 37°C

1 Pre‐warm diphasic medium to 37°C before inoculation.
2 Working in a class II biosafety cabinet, use a sterile transfer pipet to remove *Blastocystis* spp. and bacteria from the liquid phase and solid surface of the diphasic medium.
3 Transfer cells to the liquid phase of the fresh, pre‐warmed diphasic medium tubes.
4 Incubate at 37°C and subculture every 3‐4 days.
  Growth can be variable depending on ST, so frequent observation of the culture under a light microscope should be carried out.
  Results and validation from this protocol can be found in Robinson (1968).

### Support Protocol 4 Preparation of Robinson's Medium for *Blastocystis* Culturing

**Figure 8:**
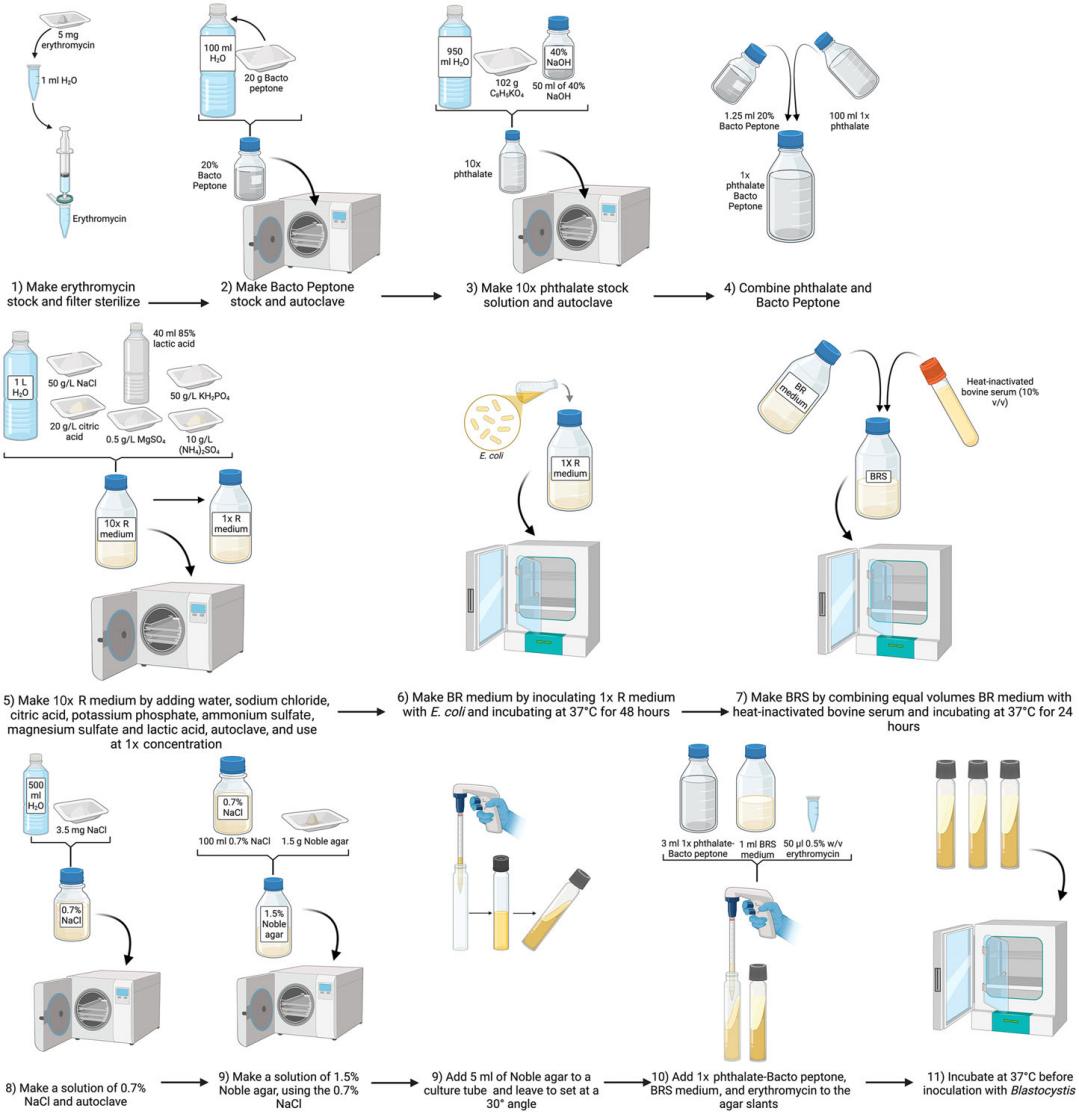
Preparation of Robinson's medium. Created in BioRender (D. Shaw, 2025; https://BioRender.com/a32z897).

Robinson's medium (Robinson, 1968) can also be used for *Blastocystis* spp., which grows well in it (Clark & Stensvold, 2016), but its use is not that widespread, likely because of its complex preparation (see Fig. 8). This medium is another whose primary usage is for *Entamoeba histolytica* culturing. It is composed of Bacto Peptone, phthalate, and BRS medium, which is inoculated with *Escherichia coli*.

***Pros and cons***

Supports robust growth of xenic *Blastocystis*, but can be labor intensive to prepare, and components such as egg can lead to batch‐to‐batch variability. The following steps make 1 L of Robinson's medium.

#### Materials

Milli‐Q‐purified water
Erythromycin
Bacto Peptone
Potassium hydrogen phthalate (C_8_H_5_KO_4_)
Sodium hydroxide (NaOH)
Citric acid
Dipotassium phosphate (KH_2_PO_4_)
Ammonium sulfate [(NH_4_)_2_SO_4_]
Magnesium sulfate heptahydrate (MgSO_4_·7H_2_O)
Lactic acid
*Escherichia coli* stock
Erythromycin

1 Prepare 0.5% (w/v) erythromycin in Milli‐Q water and filter sterilize using a 0.22‐µm‐pore‐size filter.
2 Prepare 20% (w/v) Bacto Peptone in Milli‐Q water and autoclave 30 min at 121°C, 15 psi.
3 Prepare a 10× phthalate stock solution by combining 102 g of potassium hydrogen phthalate (C_8_H_5_KO_4_), 50 ml of 40% (w/v) NaOH, and 950 ml Milli‐Q water, and then autoclave. Use at 1× concentration.
4 Prepare a phthalate/Bacto Peptone stock by combining 1.25 ml of 20% (w/v) Bacto Peptone and 100 ml of 1× phthalate.
5 Prepare a 10× R medium stock by combining 1 L Milli‐Q water, 50 g NaCl, 20 g citric acid, 50 g KH_2_PO_4_, 10 g (NH_4_)_2_SO_4_, 0.5 g MgSO_4_·7H_2_O, and 40 ml of 85% (w/v) lactic acid solution. Autoclave and use at 1× concentration.
6 Prepare BR medium by inoculating 1× R medium with *E. coli* and incubating for 48 hr at 37°C.
7 Prepare BRS medium by combining equal volumes of BR medium and heat‐inactivated bovine serum and incubating for 24 hr at 37°C.
8 Prepare 1.5% (w/v) Noble agar in 0.7% (w/v) NaCl in Milli‐Q water and autoclave.
9 Aliquot 5 ml of agar solution into tubes or bottles and leave to set at an angle.
10 Once ready to inoculate with *Blastocystis* spp., add 3 ml of 1× phthalate/Bacto Peptone, 1 ml BRS medium, and 50 µl erythromycin to the 5‐ml agar slants
11 Incubate at 37°C before use.
  Results and validation from this protocol can be found in Clark & Stensvold (2016) and Robinson (1968).

### Support Protocol 5 Preparation of Ringer's Agar Slants for *Blastocytis* Culturing

**Figure 9:**
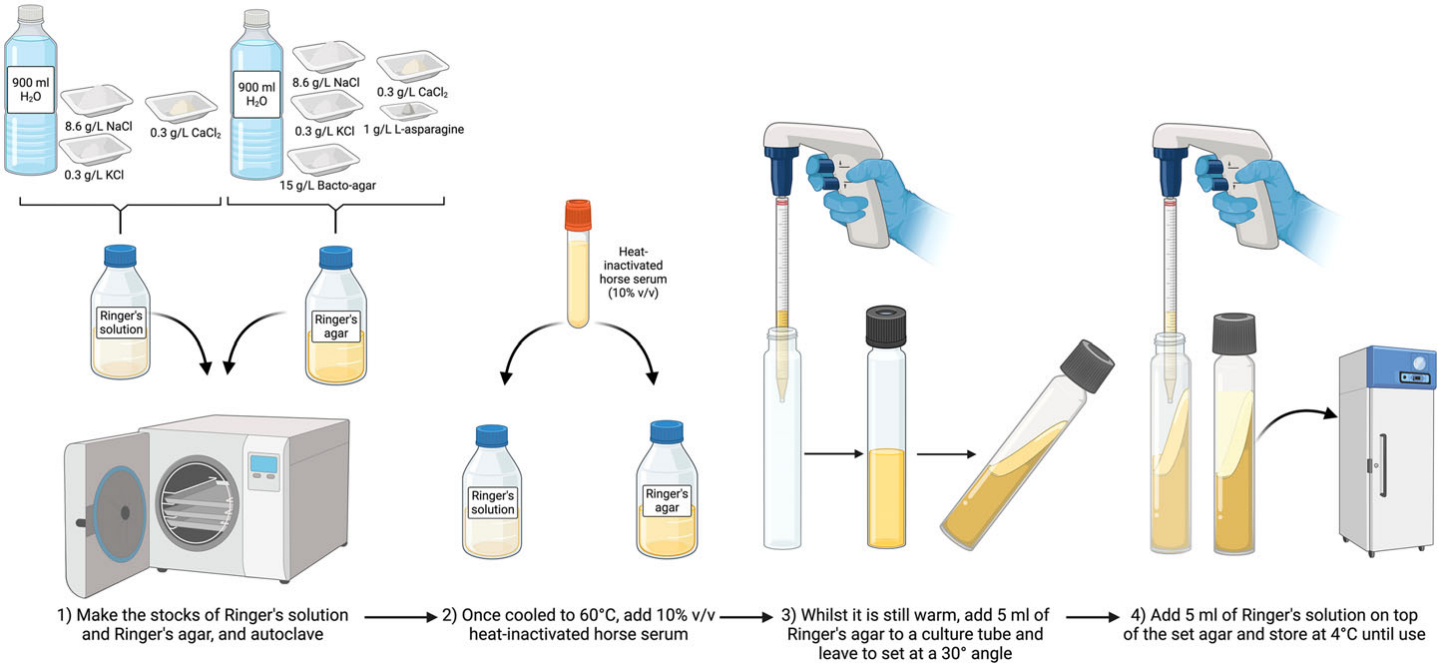
Preparation of Ringer's agar slants. Created in BioRender (D. Shaw, 2025; https://BioRender.com/u98p989).

Ringer's agar slants are composed of a solid layer of sodium chloride, calcium chloride, potassium chloride, Bacto Agar, and l‐asparagine, topped with a solution of sodium chloride, calcium chloride, and potassium chloride (see Fig. 9).

***Pros and cons***

Ringer's agar slants are simple and quick to prepare, but are not the most nutrient rich medium and cannot support axenic cultures.

#### Materials

Milli‐Q‐purified water
Sodium chloride (NaCl)
Calcium chloride (CaCl_2_)
Potassium chloride (KCl)
l‐Asparagine
1 M sodium hydroxide (NaOH)
Bacto Agar
Horse serum, heat inactivated
15‐ml Falcon tubes

1 To make Ringer's agar slants (Stenzel & Boreham, 1996), first combine the following reagents to make the solid phase:
  1 L Milli‐Q water
  8.6 g NaCl
  0.3 g CaCl_2_
  0.3 g KCl
  1 g l‐asparagine
  Adjust pH to 7.4 with 1 M NaOH, and then add:
  15 g Bacto Agar
    The solution is acidic and must be adjusted to pH 7.4 in order for *Blastocystis* spp. to grow.
2 Autoclave 30 min at 121°C, 15 psi.
3 Allow to cool to ∼60°C and add 10% (v/v) heat‐inactivated horse serum. Transfer 5‐ml aliquots into 15‐ml Falcon tubes.
  Make sure to dispense the agar into the Falcon tubes while it is still warm, to keep it from setting beforehand.
4 Leave the tubes to set at room temperature at a 30° angle.
5 For the liquid phase, combine the following reagents:
  1 L Milli‐Q water
  8.6 g NaCl
  0.3 g CaCl_2_
  0.3 g KCl
6 Autoclave 30 min at 121°C, 15 psi.
7 Once cool to touch, add 10% (v/v) heat‐inactivated horse serum.
8 Add 5 ml of the liquid phase to each tube containing the slanted solid phases.
9 Store up to 2 months at 4°C.
10 Incubate at 37°C prior before.
  Results and validation from this protocol can be found in Stenzel & Boreham (1996).

## AXENIZATION

### Basic Protocol 4 Axenization of *Blastocystis* Cultures

**Figure 10:**
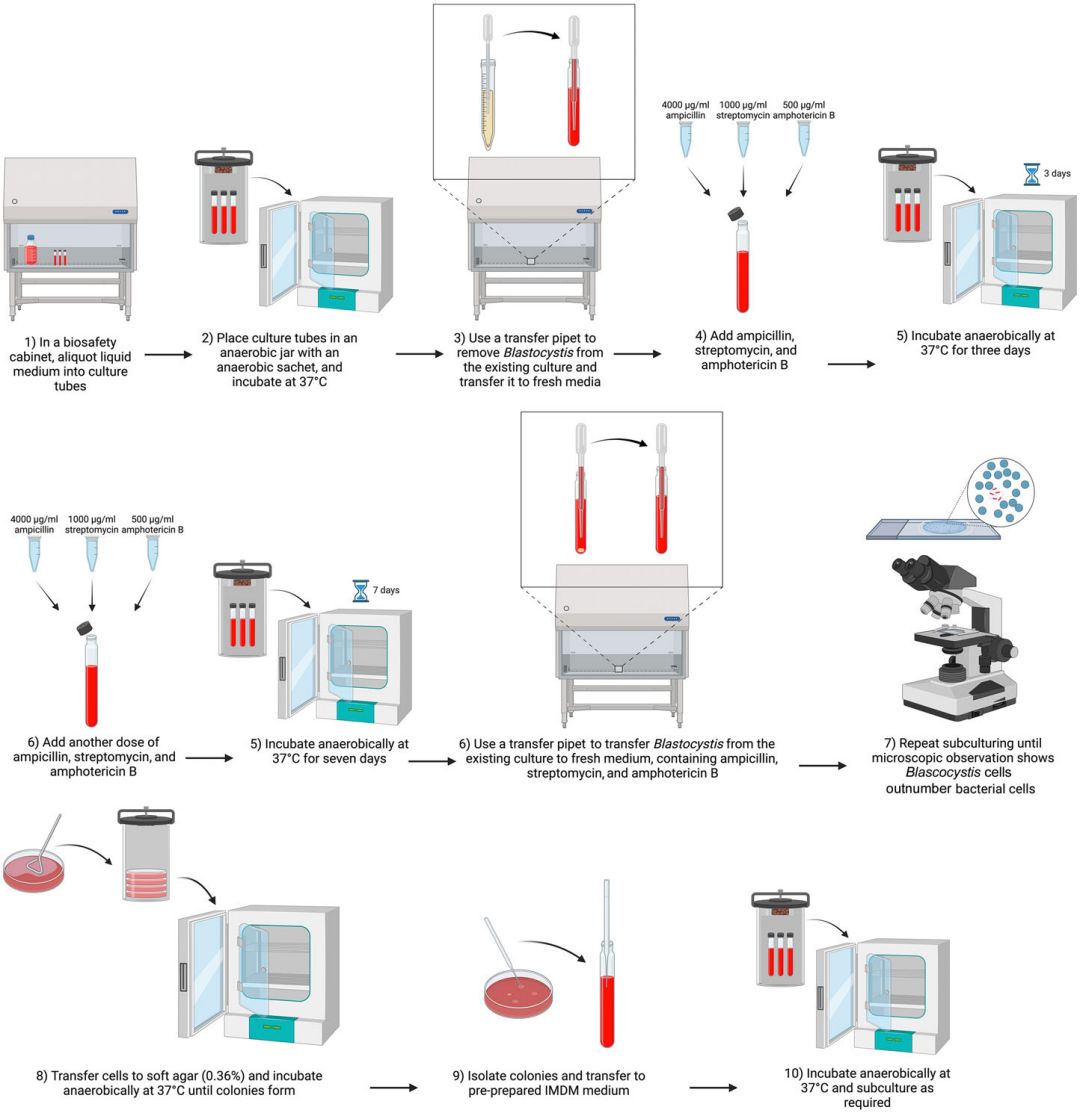
Axenization of *Blastocystis* cultures. Created in BioRender (D. Shaw, 2025; https://BioRender.com/y38z642).

*NOTE*: The following procedure should preferably be carried out in a class II biological safety cabinet.

Axenization of *Blastocystis* (see Fig. 10), that is, its culturing in the absence of any other microorganisms, remains one of the main challenges in this field. Currently, only a handful of STs have been successfully axenized and subsequently maintained in culture. These are ST1 (Zierdt et al., 1988), ST4 (Chen et al., 1997; Deng & Tan, 2022), and ST7 (Ho et al., 1993). The most commonly used method for axenization includes the elimination of bacterial populations by antibiotics (Zierdt & Williams, 1974), but it is possible that this is not always successful in removing all bacteria (Ng & Tan, 1999). It is therefore recommended to follow this with clonal growth on soft (Tan, Singh, Thong, et al., 1996) or solid (Tan et al., 2000) agar, where individual colonies of *Blastocystis* can be isolated.

#### Materials

Xenic *Blastocystis* spp. culture
Fresh medium
Ampicillin
Streptomycin
Amphotericin B
IMDM soft (0.36%) agar (prepared as in Basic Protocol 7)
Pre‐reduced liquid IMDM (see Basic Protocol 6, steps 1‐5), containing 10% (v/v) heat‐inactivated horse serum

Class II biosafety cabinet
Incubator, 37°C
Anaerobic chamber

1 Pre‐warm and pre‐reduce fresh medium to 37°C before inoculation.
  To pre‐reduce medium, place in anaerobic conditions for 24 hr before use.
2 Working in a class II biosafety cabinet, inoculate fresh medium with a xenic culture of *Blastocystis* (Basic Protocol 2).
3 Add an antimicrobial mixture of 4000 µg/ml ampicillin, 1000 µg/ml streptomycin, and 500 µg/ml amphotericin B.
4 Incubate at 37°C under anaerobic conditions.
  For anaerobic culturing, we recommend using an anaerobic jar system, such as the Anoxomat III Anaerobic Jar System or the Oxoid AnaeroJar with BD GasPak EZ sachets
5 After 3 days, add another 4000 µg/ml ampicillin, 1000 µg/ml streptomycin, and 500 µg/ml amphotericin B.
6 Incubate at 37°C for 7 days.
7 After 7 days, subculture to fresh medium (see Basic Protocol 2) with 4000 µg/ml ampicillin, 1000 µg/ml streptomycin, and 500 µg/ml amphotericin B.
8 Repeat subculturing until microscopic observation shows that *Blastocystis* cells outnumber bacterial cells.
9 Transfer *Blastocystis* cells to IMDM soft (0.36%) agar (see Basic Protocol 7).
10 Once colonies are visible (after ∼7 days), isolate colonies using a Pasteur pipet and transfer to pre‐reduced liquid IMDM containing 10% (v/v) heat‐inactivated horse serum.
  Results and validation from this protocol can be found in Chen et al. (1997), Deng & Tan (2022), Ho et al. (1993), Ng & Tan (1999), Tan et al. (2000), Tan, Singh, Thong, et al. (1996), Zierdt et al. (1988), and Zierdt & Williams (1974).

## MEDIA AND CULTURING METHODS FOR AXENIC CULTURES IN DIPHASIC MEDIA

### Basic Protocol 5 Establishment of Axenic *Blastocystis* Culture in Diphasic Medium

**Figure 11:**
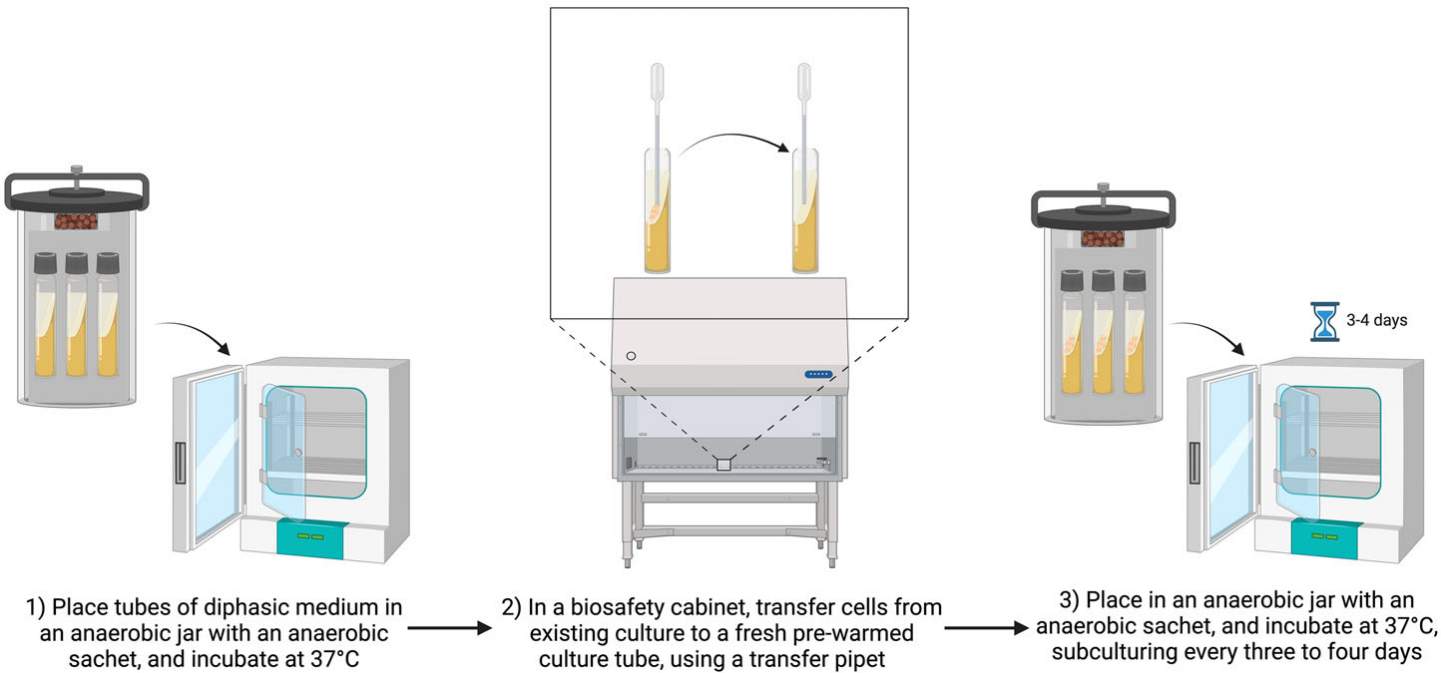
Culturing of axenic *Blastocystis* subtypes in diphasic medium. Created in BioRender (D. Shaw, 2025; https://BioRender.com/x44u072).

The following procedure should preferably be carried out in a class II biological safety cabinet (Fig. 11).

#### Materials

Fresh diphasic medium
Ampicillin
Streptomycin
Amphotericin B

Class II biosafety cabinet
Incubator suitable for anaerobic culture, 37°C

1 Pre‐reduce diphasic medium and pre‐warm to 37°C before inoculation.
  To pre‐reduce medium, place in anaerobic conditions for 24 hr before use.
2 Working in a class II biosafety cabinet, use a sterile transfer pipet to remove *Blastocystis* spp. from the liquid phase and solid surface of the diphasic medium.
3 Transfer cells to the liquid phase of the fresh, pre‐warmed diphasic medium tubes.
4 Incubate at 37°C anaerobically and subculture every 3‐4 days.
  To culture anaerobically, it is recommended to use an anaerobic jar system, such as the Anoxomat III Anaerobic Jar System or the Oxoid AnaeroJar with BD GasPak EZ sachets
  Growth can be variable depending on ST, so frequent observation of the culture under a light microscope should be carried out.
  Results and validation from this protocol can be found in Lanuza et al. (1996).

### Support Protocol 6 Preparation of Boeck and Drbohlav's Locke‐Egg Serum (LES) Medium for *Blastocystis* Culturing

**Figure 12:**
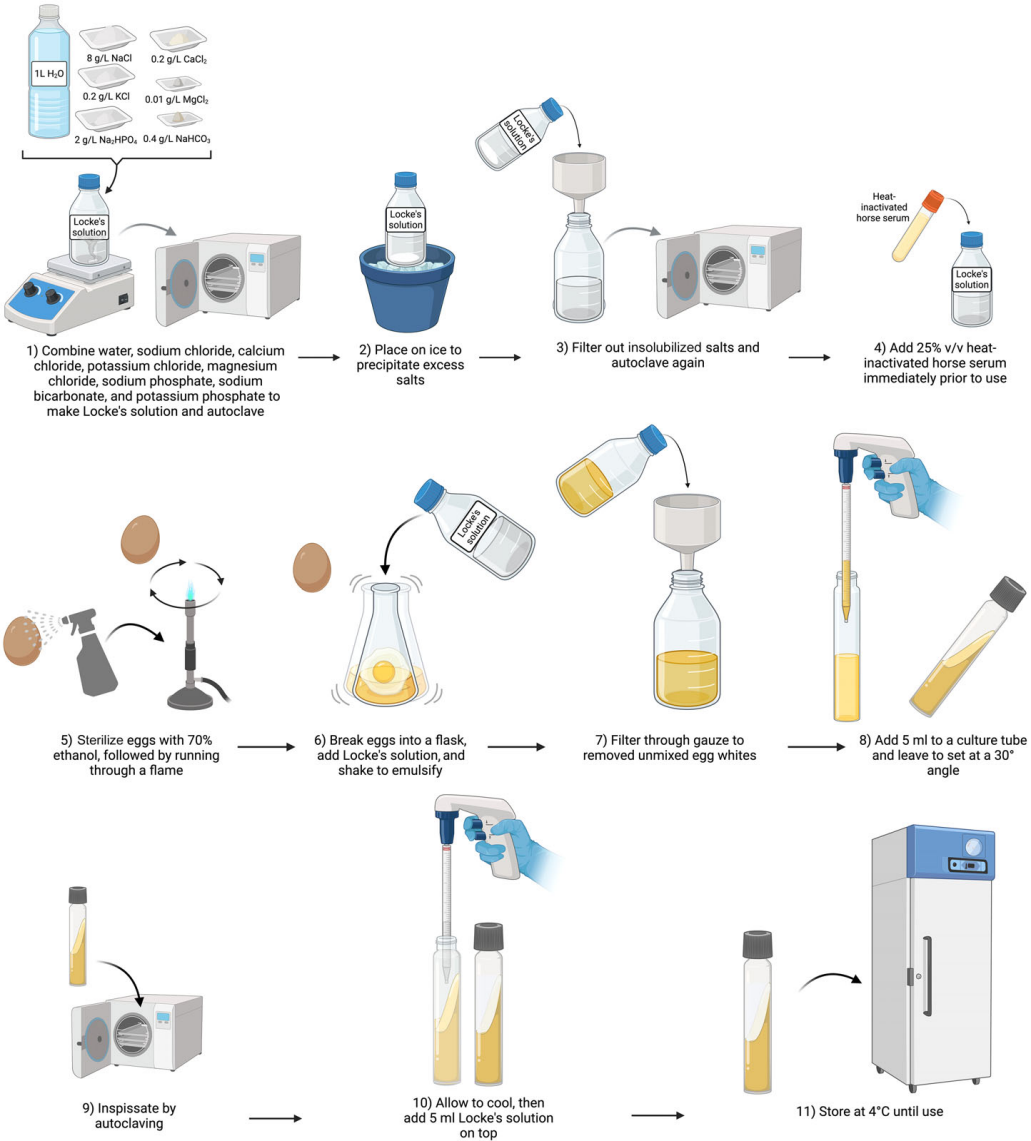
Preparation of Boeck and Drbohlav's Locke‐egg serum (LES) medium. Created in BioRender (D. Shaw, 2025; https://BioRender.com/u13l909).

Boeck and Drbohlav's Locke‐egg serum (LES) medium (see Fig. 12) was also first used to establish cultures of *Entamoeba histolytica* (Boeck & Drbohlav, 1925). This medium consists of a solution of sodium chloride, calcium chloride, potassium chloride, magnesium chloride, sodium phosphate (dibasic), sodium bicarbonate, and potassium phosphate (monobasic) combined with whole eggs. This egg mixture undergoes inspissation to increase its viscosity and form the solid phase. It is then topped with Locke's solution, supplemented with heat‐inactivated horse serum.

***Pros and cons***

Boeck and Drbohlav's LES medium is rich in nutrients and effective for isolation of axenic cultures from fecal samples, but is labor intensive to prepare, requiring inspissation, and contains components such as eggs, which can lead to batch‐to‐batch variability.

#### Materials

Milli‐Q‐purified water
Sodium chloride (NaCl)
Calcium chloride (CaCl_2_)
Potassium chloride (KCl)
Magnesium chloride (MgCl_2_)
Disodium phosphate (Na_2_HPO_4_)
Sodium bicarbonate (NaHCO_3_)
Monopotassium phosphate (KH_2_PO_4_)
Horse serum, heat inactivated
70% (v/v) ethanol

Whatman filter paper, grade 1
Gauze

1 To prepare Locke's solution, combine the following reagents and autoclave 30 min at 121°C at 15 psi:
  1 L Milli‐Q water
  8 g NaCl
  0.2 g CaCl_2_
  0.2 g KCl
  0.01 g MgCl_2_
  2 g Na_2_HPO_4_
  0.4 g NaHCO_3_
  0.3 g KH_2_PO_4_
2 Place on ice to allow precipitation, filter out insolubilized salts using grade 1 Whatman paper, and then re‐autoclave.
3 Prepare the egg slants (Clark & Diamond, 2002; CDC, 1948):
  a. Sterilize eggs by spraying each one with 70% ethanol and running it through a flame.
  b. Break whole eggs into a flask, add 12.5 ml Locke's solution (from step 2) for every whole egg (∼45 ml) and shake to emulsify.
  c. Filter the mixture through gauze to remove all unmixed egg white.
  d. Transfer 5 ml to a sterile culture tube and apply a vacuum for 1 hr to remove air bubbles.
4 Let tube rest at a 30° angle to form a slant and cook at 120°C for 30 min.
5 Wait until cool, add 5 ml sterile Locke's solution (from step 2) on top of the solid phase, and then inspissate by autoclaving.
6 Store at 4°C and add 25% (v/v) heat‐inactivated horse serum before use.
  Results and validation from this protocol can be found in Boeck & Drbohlav (1925), Clark & Diamond (2002), and CDC (1948).

### Support Protocol 7 Preparation of Boeck and Drbohlav's Diphasic Modified Medium (BDMM) for *Blastocystis* Culturing

**Figure 13:**
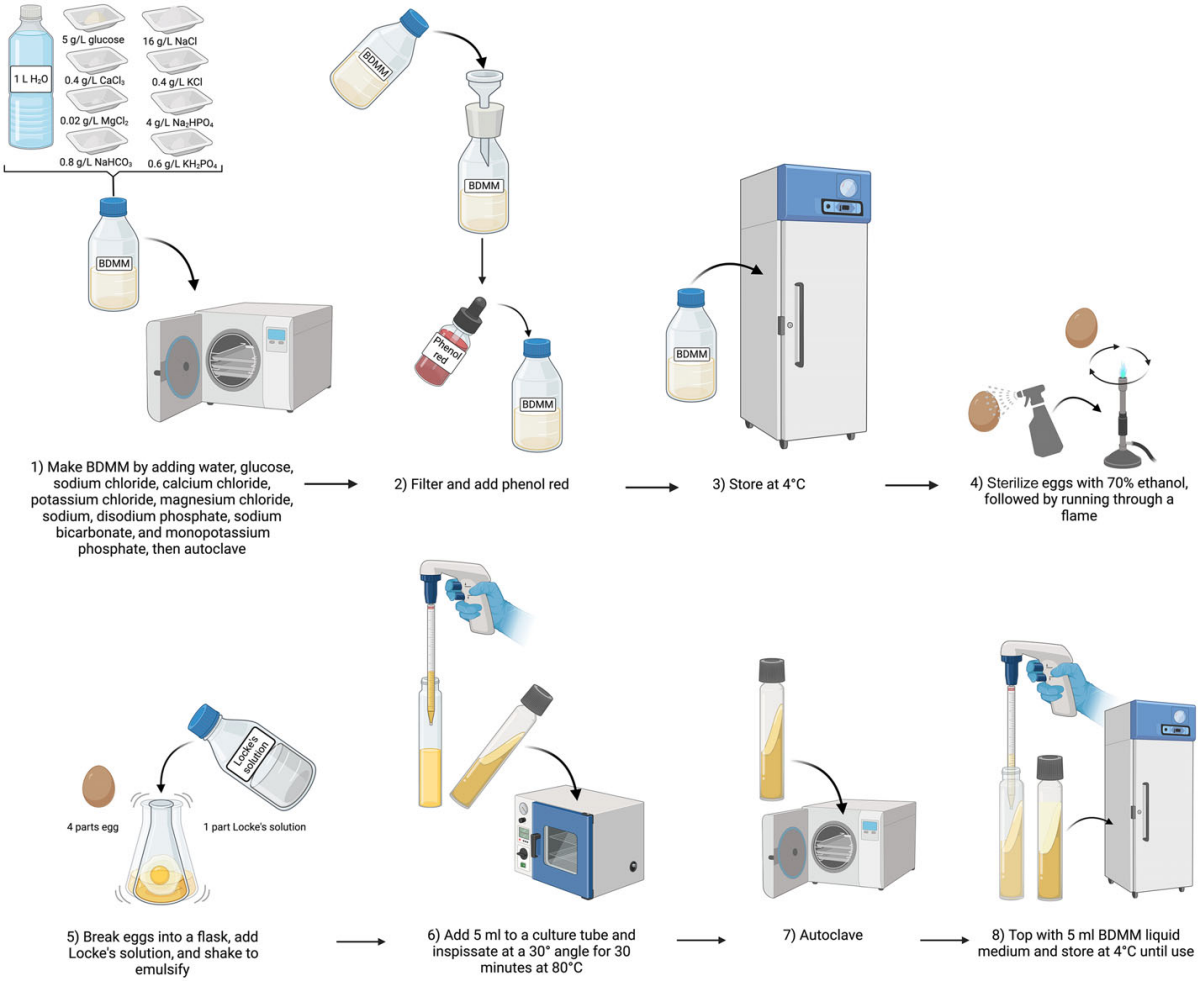
Preparation of Boeck and Drbohlav's diphasic modified medium (BDMM). Created in BioRender (D. Shaw, 2025; https://BioRender.com/w55n055).

In 1996, Lanuza et al. published their use of a modified recipe of Boeck and Drbohlav's LES medium (BDMM; see Fig. 13), which contained the same ingredients as the standard recipe at twice the original concentration, as well as glucose (Lanuza et al., 1996).

***Pros and cons***

BDMM is rich in nutrients and effective for growth of axenic cultures, but is labor intensive to prepare, requiring inspissation, and contains components such as eggs that can lead to batch‐to‐batch variability.

#### Materials

Milli‐Q‐purified water
Glucose
Sodium chloride (NaCl)
Calcium chloride (CaCl_2_)
Potassium chloride (KCl)
Magnesium chloride (MgCl_2_)
Disodium phosphate (Na_2_HPO_4_)
Sodium bicarbonate (NaHCO_3_)
Monopotassium phosphate (KH_2_PO_4_)
Phenol red
Locke's solution (see Support Protocol 6)
70% (v/v) ethanol

Whatman paper, grade 1
Anaerobic chamber

1 To make BDMM solution, combine the following reagents:
  1 L Milli‐Q water
  5 g glucose
  16 g NaCl
  0.4 g CaCl_2_
  0.4 g KCl
  0.02 g MgCl_2_
  4 g Na_2_HPO_4_
  0.8 g NaHCO_3_
  0.6 g KH_2_PO_4_
2 Autoclave 30 min at 121°C, 15 psi.
3 Filter through grade 1 Whatman paper to remove insolubilized salts. Add 0.02% (w/v) phenol red.
4 Autoclave again and store at 4°C.
5 For the solid phase of BDMM, first sterilize eggs by spraying each with 70% ethanol and running it through a flame.
6 Combine the whole eggs with Locke's solution (see Support Protocol 6) at 4:1 (v/v) and homogenize.
7 Transfer 5 ml of this mixture to sterile culture tubes and inspissate at a 30° angle for 30 min at 80°C.
8 Autoclave 30 min at 121°C, 15 psi.
9 Top with 5 ml of the BDMM liquid medium from step 4.
10 Store at 4°C until ready for use.
11 Pre‐reduce in an anaerobic chamber before use.
  Results and validation from this protocol can be found in Lanuza et al. (1996).

## MEDIUM AND CULTURING METHODS FOR AXENIC CULTURES IN LIQUID MEDIUM

**Figure 14:**
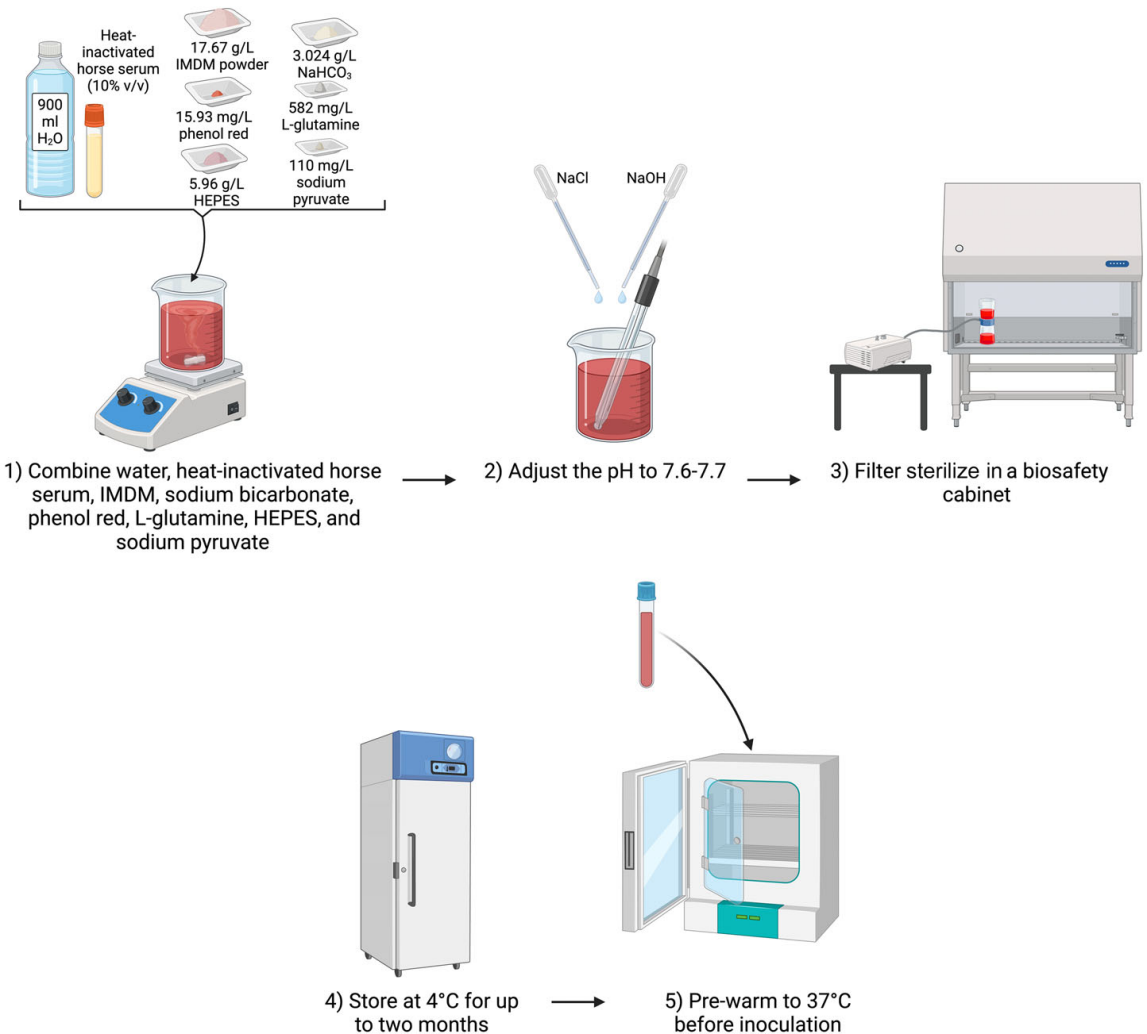
Preparation of Iscove's modified Dulbecco's medium (IMDM). Created in BioRender (D. Shaw, 2025; https://BioRender.com/x27r491).

Very few STs of *Blastocystis* spp. have been successfully axenized. In 1993, Ho et al. optimized the axenic culturing of *Blastocystis* spp. in Iscove's modified Dulbecco's medium (IMDM), supplemented with 10% (v/v) heat‐inactivated horse serum (Ho et al., 1993). IMDM medium (see Fig. 14) used for cultivation of *Blastocystis* spp. must contain l‐glutamine, sodium bicarbonate, HEPES buffer, phenol red, and sodium pyruvate. Medium should be pre‐reduced prior to inoculation with *Blastocystis* spp. and then incubated inside anaerobic jars to maintain an anaerobic environment.

### Basic Protocol 6 Establishment of Axenic *Blastocystis* Cultures in Iscove's Modified Dulbecco's Medium (IMDM)

***Pros and cons***

**Figure 15:**
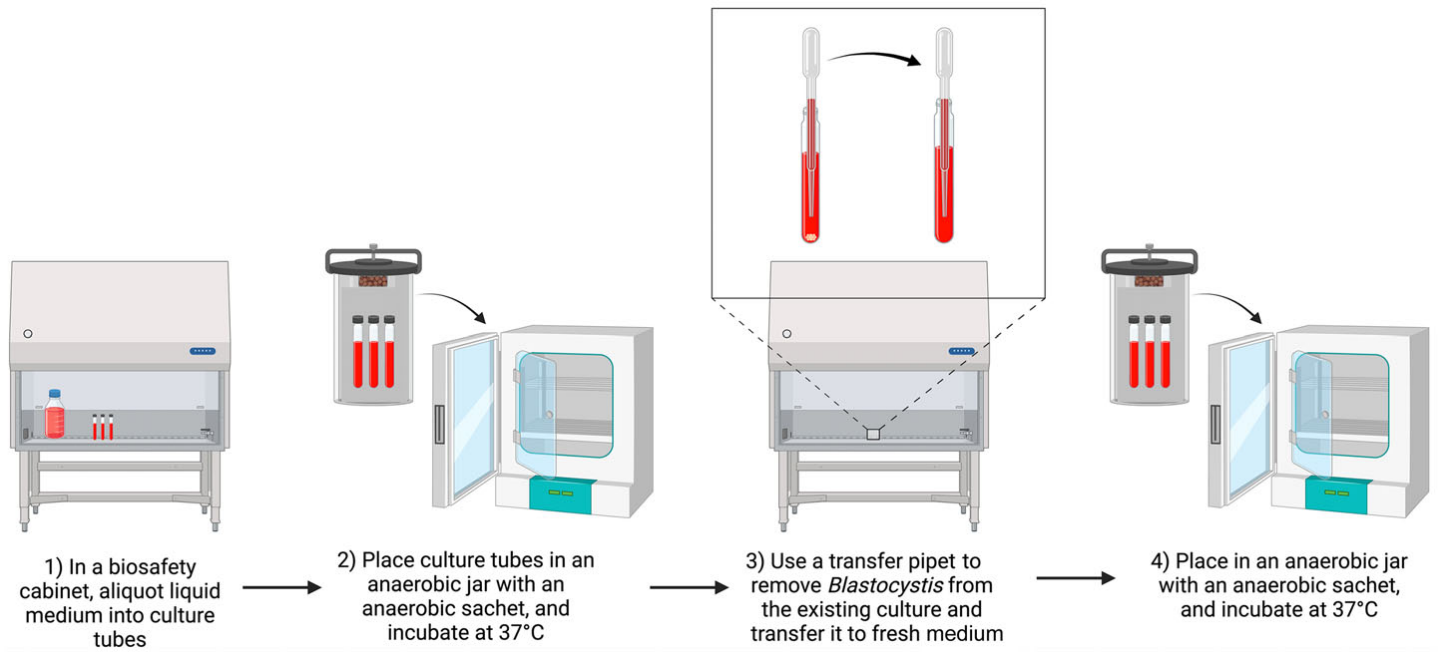
Culturing of axenic *Blastocystis* subtypes in liquid medium. Created in BioRender (D. Shaw, 2025; https://BioRender.com/a49r646).

The following procedure (Fig. 15) should preferably be carried out in a class II biosafety cabinet. Note that because it contains HEPES, IMDM is a buffered medium and can easily be supplemented for growth of axenic cultures; however, it can be more expensive that other culture media.

#### Materials

Milli‐Q‐purified water
Horse serum, heat inactivated
IMDM powdered medium
*If not included in IMDM powder*:
l‐Glutamine
Sodium bicarbonate (NaHCO_3_)
4‐(2‐Hydroxyethyl)‐1‐piperazine ethanesulfonic acid (HEPES)
Phenol red
Sodium pyruvate
10,000 U/ml penicillin/10 mg/ml streptomycin (optional)
NaOH and/or HCl, for pH adjustment

0.22‐µm‐pore‐size filter
14‐ml round‐bottom culture tubes with vented caps
Anaerobic jar with anaerobic‐gas‐generating sachets (e.g., Anoxomat III Anaerobic Jar System or the Oxoid AnaeroJar with BD GasPak EZ sachets)

##### Medium preparation

1 To make 1 L of complete IMDM medium, add the following reagents and filter sterilize using a 0.22‐µm‐pore‐size vacuum filter (do not autoclave IMDM):
  900 ml Milli‐Q water
  100 ml heat‐inactivated horse serum
  17.67 g IMDM powder
2 *OPTIONAL*: If your manufacturer's IMDM powder does not contain any of the following, add these to the solution:
  3.024 g/L NaHCO_3_
  15.93 mg/L phenol red
  582 mg/L l‐glutamine
  5.96 g/L HEPES
  110 mg/L sodium pyruvate
    There are also many pre‐prepared IMDM powders pre‐supplemented with some or all of these components, so this step may not be necessary.
*OPTIONAL*: Add 1% (v/v) 10,000 U/ml penicillin/10 mg/ml streptomycin.
3 The pH of the final solution may vary depending on the source of horse serum. Adjust pH using NaOH and HCl to achieve a pH of 7.6‐7.7.
4 Filter using a 0.22‐µm‐pore‐size filter and store at 4°C until use.

##### Inoculation and culturing

Before inoculation with *Blastocystis* spp., the IMDM medium must be pre‐reduced (by being placed in anaerobic conditions) and pre‐warmed at 37°C.

5 Transfer 8 ml complete IMDM medium to 14‐ml round‐bottom culture tubes, closing the cap to the vented position.
6 Place the tubes in an anaerobic jar alongside an anaerobic‐gas‐generating sachet to produce a hypoxic environment.
7 Incubate anaerobically at 37°C for 24 hr.
  For anaerobic culturing, we recommend using an anaerobic jar system, such as the Anoxomat III Anaerobic Jar System or the Oxoid AnaeroJar with BD GasPak EZ sachets.
8 Inoculate each tube with the visual sediment from the previous culture of *Blastocystis* spp. and culture anaerobically at 37°C.
9 Subculture into fresh medium every 4‐5 days.
  Growth can be variable depending on ST, so frequent observation of the culture should be carried out.
  Results and validation from this protocol can be found in Ho et al. (1993).

## MEDIA AND CULTURING METHODS FOR AXENIC CULTURES IN SEMI‐SOLID AND SOLID MEDIA

Monophasic solid or semi‐solid media are less commonly used than liquid media, although it is possible to culture colonies of *Blastocystis* spp. in this way.

### Basic Protocol 7 Establishment of Axenic *Blastocystis* Cultures in Soft IMDM Agar

**Figure 16:**
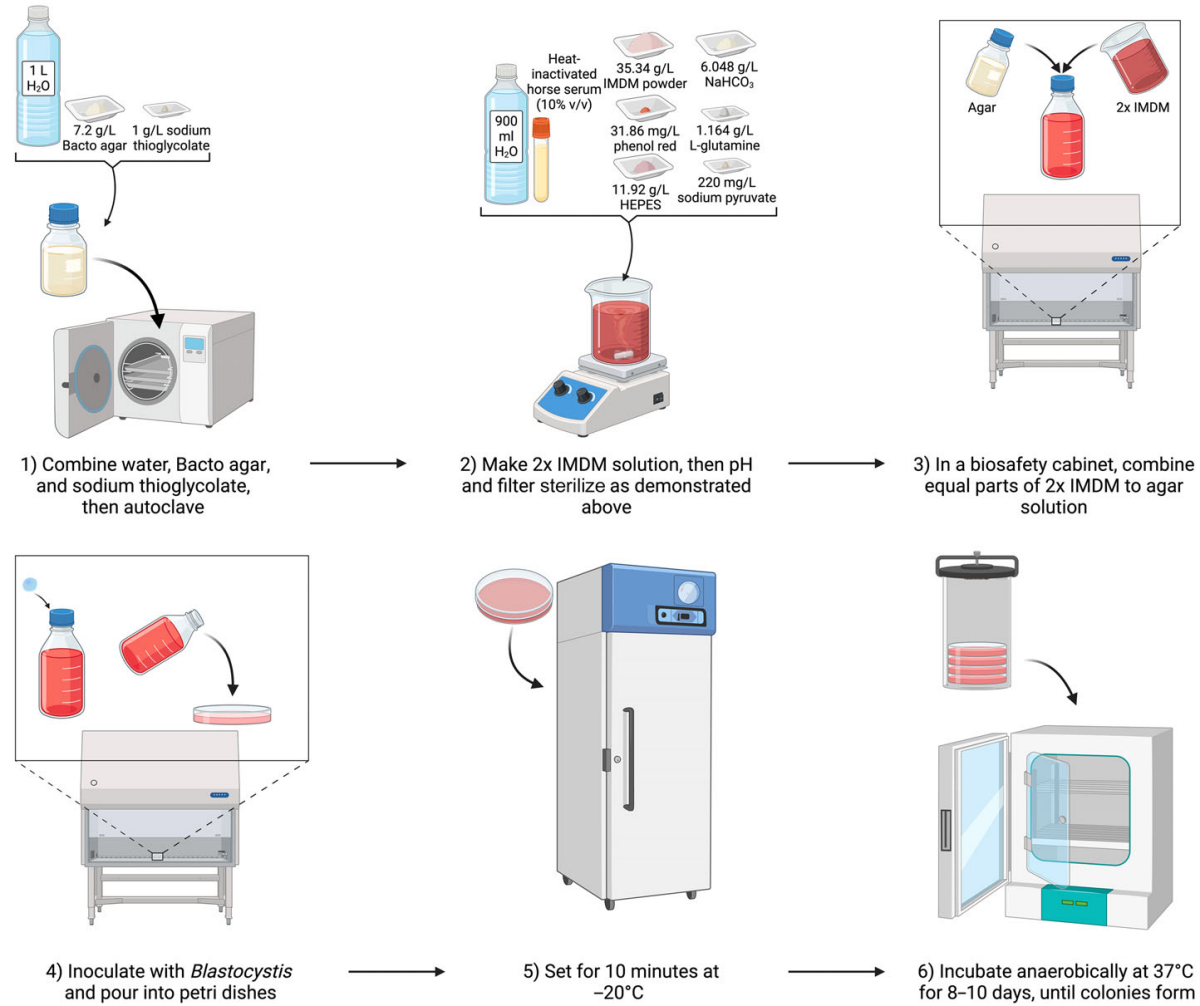
Culturing of axenic *Blastocystis* subtypes in IMDM 0.36% soft agar. Created in BioRender (D. Shaw, 2025; https://BioRender.com/o40p601).

In 1996, Tan et al. published the first description of axenic *Blastocystis* spp. colony formation on soft IMDM with 0.36% (w/v) agar (Tan, Singh, Yap, et al., 1996). In the same year, they updated the protocol (see Fig. 16) as they found that adding the reducing agent sodium thioglycolate to the soft agar improved colony yield of biconvex disc‐shaped colonies, which could have previously been hindered by time needed to reach anaerobiosis after plating (Tan, Singh, Thong, et al., 1996).

***Pros and cons***

IMDM soft (0.36%) agar is optimal for anaerobic culturing due to the addition of sodium thioglycolate, supporting axenic colony formation; however, growth on this medium is slow.

*NOTE*: The following procedure should preferably be carried out in a class II biological safety cabinet.

#### Materials

Milli‐Q‐purified water
Bacto Agar
Sodium thioglycolate
2× liquid IMDM medium
Horse serum, heat inactivated
*Blastocystis* culture in liquid medium
Class II biosafety cabinet
Anaerobic jar with anaerobic‐gas‐generating sachets (e.g., Anoxomat III Anaerobic Jar System or the Oxoid AnaeroJar with BD GasPak EZ sachets)

1 To prepare IMDM soft agar, first make the agar by combining the following reagents:
  1 L Milli‐Q water
  7.2 g Bacto Agar
  1 g sodium thioglycolate
  Autoclave 30 min at 121°C, 15 psi.
2 Working in a class II biosafety cabinet, add 1 part agar to 1 part 2× IMDM medium (adapted from Basic Protocol 6), along with 10% (w/v) heat‐inactivated horse serum, and inoculate with all visual sediment of *Blastocystis* spp. from culture.
3 Pour ~20 ml/dish into 90‐mm petri dishes and allow to set for 10 min at –20°C.
4 Incubate at 37°C in an anaerobic jar for 8‐10 days, until colonies form.
  For anaerobic culture, we recommend using an anaerobic jar system, such as the Anoxomat III Anaerobic Jar System or the Oxoid AnaeroJar with BD GasPak EZ sachets.
  Growth can be variable depending on ST, so frequent observation of the culture under a light microscope should be carried out.
  Results and validation from this protocol can be found in Tan, Singh, Yap, et al. (1996).

### Basic Protocol 8 Establishment of Axenic *Blastocystis* Cultures on Solid IMDM Agar

**Figure 17:**
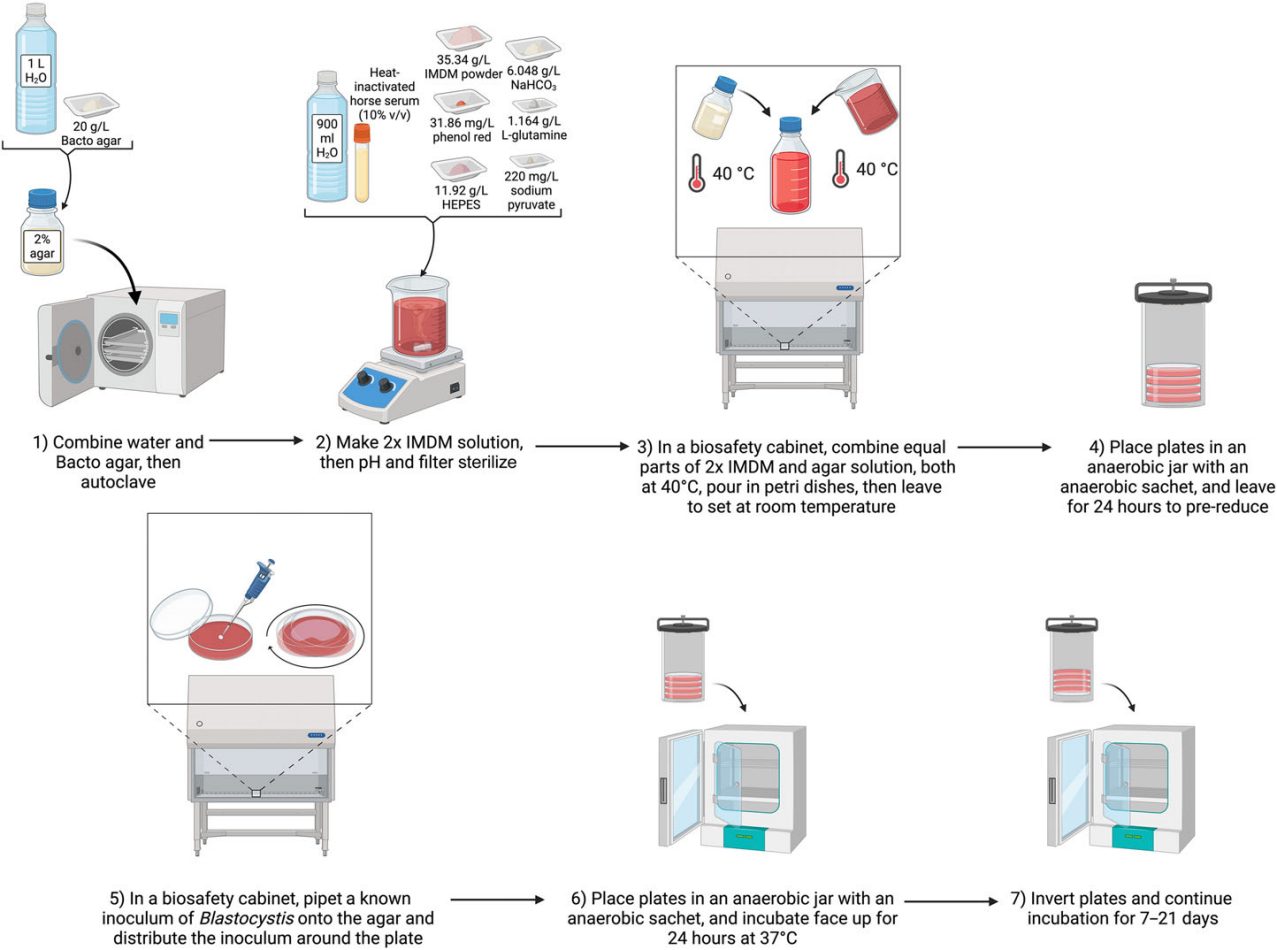
Culturing of axenic *Blastocystis* subtypes in IMDM solid agar. Created in BioRender (D. Shaw, 2025; https://BioRender.com/w71 × 738).

*NOTE*: The following procedure should preferably be carried out in a class II biological safety cabinet.

Tan et al. later successfully grew *Blastocystis* spp. colonies on solid IMDM agar (Tan et al., 2000). This method does not require the addition of sodium thioglycolate, as the plates are pre‐reduced before inoculation with *Blastocystis* spp. (see Fig. 17).

***Pros and cons***

IMDM solid agar (2%) does not require chemical reducers, instead relying on being pre‐reduced in an anaerobic chamber; however, this medium requires an even longer incubation time for colony formation than soft agar.

#### Materials

Milli‐Q‐purified water
Bacto Agar
2× liquid IMDM medium, pre‐warmed to 40°C
Horse serum, heat inactivated, pre‐warmed to 40°C
*Blastocystis* culture in liquid medium

Petri dishes
Class II biosafety cabinet
Anaerobic jar with anaerobic‐gas‐generating sachets (e.g., Anoxomat III Anaerobic Jar System or the Oxoid AnaeroJar with BD GasPak EZ sachets)

##### Medium preparation

1 To prepare solid IMDM agar, first make a 2% (w/v) agar stock by combining the following reagents:
  1 L Milli‐Q water
  20 g Bacto Agar
2 Autoclave 30 min at 121°C, 15 psi.
3 Once cooled, add equal parts of 2% agar to 2× IMDM supplemented with 10% (v/v) heat‐inactivated horse serum, both at 40°C.
4 Pour ~20 ml/dish into 90‐mm petri dishes and leave to solidify at room temperature.

##### Inoculation and culturing

5 Transfer to an anaerobic chamber to pre‐reduce for 24 hr before inoculation with *Blastocystis* spp.
  To pre‐reduce medium, place in anaerobic conditions for 24 hr before use.
6 Working in a class II biosafety cabinet, pipet an inoculation‐loop‐sized amount of *Blastocystis* spp. onto the solid agar and gently rock to distribute the protozoa around the plate.
  Alternatively, plating using the classical streaking technique employed in bacteriology may also be used to obtain single colonies from undefined initial inoculum concentrations.
7 Incubate in an anaerobic chamber for 24 hr at 37°C, face up to allow the protozoa to colonize the agar surface.
8 After 24 h, invert the plates and continue to incubate in an anaerobic chamber at 37°C for 7‐21 days.
  For anaerobic culture, we recommend using an anaerobic jar system, such as the Anoxomat III Anaerobic Jar System or the Oxoid AnaeroJar with BD GasPak EZ sachets.
  Growth can be variable depending on ST, so frequent observation of the culture under a light microscope should be carried out.
  Results and validation from this protocol can be found in Tan et al. (2000).

### Basic Protocol 9 Optimized Method for Establishing Axenic *Blastocystis* Cultures on Solid IMDM Agar

**Figure 18:**
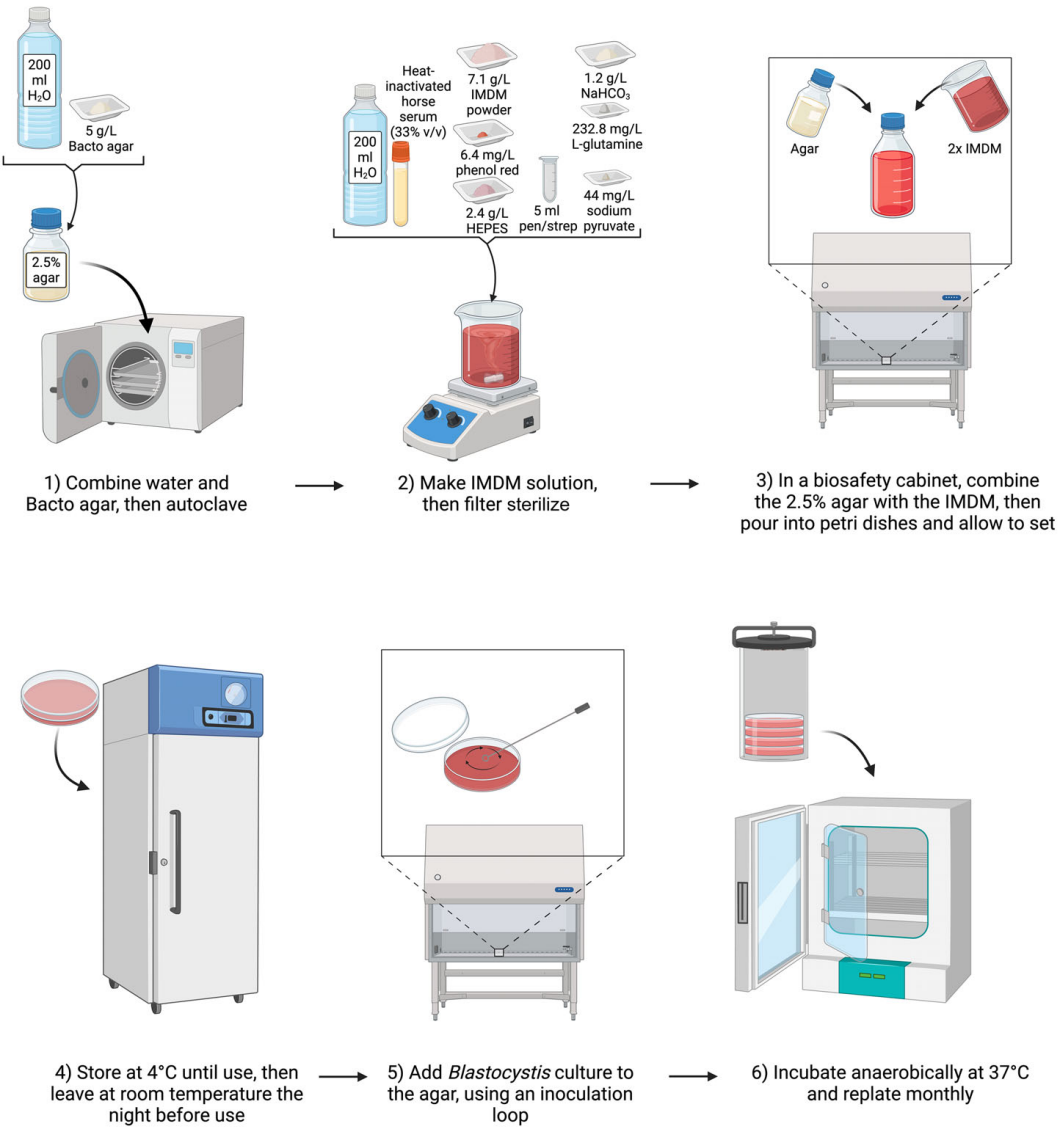
Culturing of axenic *Blastocystis* subtypes in IMDM solid agar (specifically optimized for ST8). Created in BioRender (D. Shaw, 2025; https://BioRender.com/z01w572).

*NOTE*: This method is not widely used in the literature, and any deviations from the standard protocol should be optimized for the lab that will be using it.

*NOTE*: The following procedure should preferably be carried out in a class II biological safety cabinet.

Some groups have optimized the standard protocol for use with specific subtypes. For example, the IMDM solid agar recipe has been optimized for culturing ST8 (see Fig. 18) using the following protocol, which was established for an axenic culture of ST8 (E. Viscogliosi lab, personal communication).

***Pros and cons***

IMDM solid agar (2.5%) has a higher percentage of agar than other solid media, making it easier to inoculate; however, is specifically optimized for ST8 and so may not produce reliable results for other STs.

#### Materials

Bacto Agar
Milli‐Q‐purified water
IMDM powdered medium
Horse serum, heat inactivated
*Blastocystis* culture in liquid medium
10,000 U/ml penicillin/10 mg/ml streptomycin

Water bath, 50°C
0.22‐µm‐pore‐size vacuum filter
Class II biosafety cabinet
Petri dishes
Anaerobic jar with anaerobic‐gas‐generating sachets (e.g., Anoxomat III Anaerobic Jar System or the Oxoid AnaeroJar with BD GasPak EZ sachets)

##### Medium preparation

1 First, prepare a 2.5% (w/v) agar stock by combining:
  25 g Bacto Agar
  1 L Milli‐Q water
2 Autoclave 30 min at 121°C, 15 psi.
3 Keep mixture warm in a water bath at 50°C to prevent it from solidifying.
4 Prepare the IMDM portion of the medium by combining:
  7.1 g IMDM powder
  200 ml Milli‐Q water
  100 ml heat‐inactivated horse serum
  5 ml of 10,000 U/ml penicillin/10 mg/ml streptomycin
5 Filter sterilize using a 0.22‐µm‐pore‐size vacuum filter.
6 Remove agar preparation from the water bath and allow to cool for 5 min.
7 Preferably working in a class II biosafety cabinet, add the IMDM solution to the agar preparation.
  Always mix the solutions in the agar bottle to avoid having the agar stick to the sides.
8 Pour ~20 ml/dish of the solution into 90‐mm petri dishes and allow to set inside the category II biosafety cabinet.
9 Store at 4°C until use.

##### Inoculation and culturing

**Figure 19:**
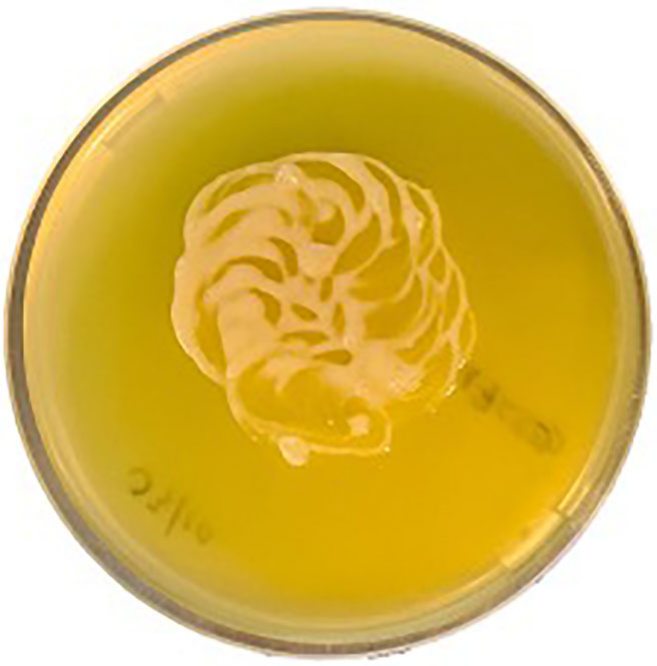
ST8 culture grown on IMDM agar. Photograph courtesy of Constance Denoyelle.

10 The night before use, place the petri dish at room temperature in the biosafety cabinet.
11 The next day, using an inoculation loop, transfer *Blastocystis* spp. culture to the agar, making small circular motions.
12 Incubate at 37°C in an anaerobic chamber with an anaerobic sachet, replating monthly.
  Results from this protocol are presented below in Figure 19.

### Basic Protocol 10 Establishment of Axenic *Blastocystis* Cultures in Semi‐Solid Locke's Agar

Later, in 2007, Valido and Rivera published an adapted recipe for soft Locke's agar (see Fig. 19), with a final concentration of 0.7% (w/v) agar, which resulted in biconvex disc‐shaped colonies as seen previously (Valido & Rivera, 2007).

*NOTE*: The following procedure should preferably be carried out in a class II biological safety cabinet.

***Pros and cons***

Semi‐solid Locke's agar (0.7%) supports the formation of distinct biconvex disc‐shaped colonies; however, because of its consistency, it can be difficult to handle.

#### Materials

Milli‐Q‐purified water
Bacto Agar
Sodium thioglycolate
Locke's solution (see Support Protocol 6, step 1), sterilized
Horse serum, heat inactivated
*Blastocystis* culture in liquid medium

Class II biosafety cabinet
Petri dishes
Anaerobic jar with anaerobic‐gas‐generating sachets (e.g., Anoxomat III Anaerobic Jar System or the Oxoid AnaeroJar with BD GasPak EZ sachets)

**Figure 20:**
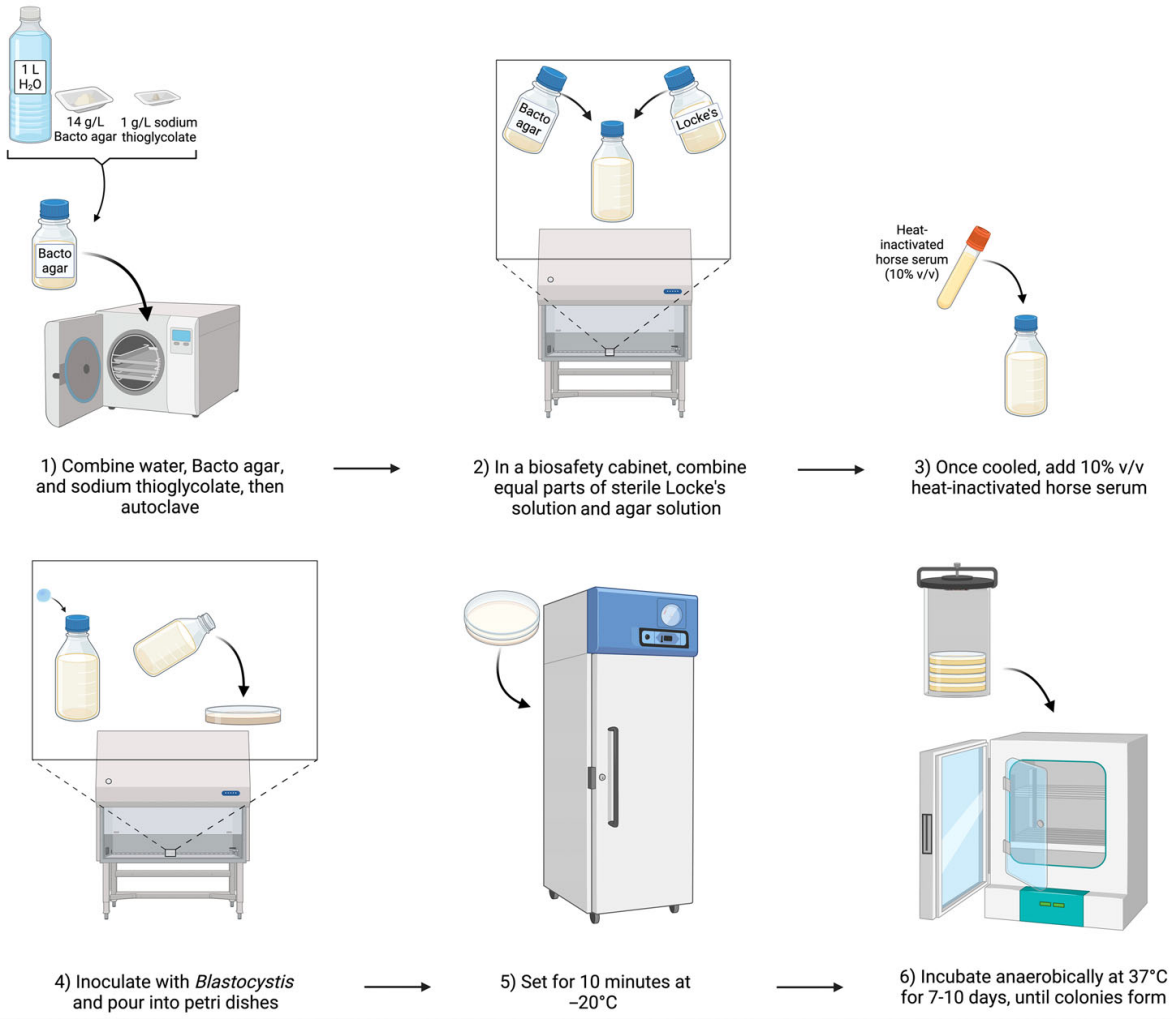
Culturing of axenic *Blastocystis* subtypes in semi‐solid Locke's agar. Created in BioRender (D. Shaw, 2025; https://BioRender.com/g82t511).

1 To prepare semi‐solid Locke's agar, first make a 0.7% (w/v) agar stock by combining the following reagents (Fig. 20):
  1 L Milli‐Q water
  14 g Bacto Agar
  1 g sodium thioglycolate

2 Autoclave at 121°C for 30 min at 15 psi.
3 Add in equal parts to sterilized Locke's solution.
4 Allow to cool to 40°C and add 10% (v/v) heat‐inactivated horse serum.
5 Working in a class II biosafety cabinet, inoculate with *Blastocystis* spp. and pour ~20 ml/dish into 90‐mm petri dishes.
6 Cool for 10 min at –20°C to allow the agar to set.
7 Transfer the plates to an anaerobic chamber and incubate at 37°C for 7‐10 days.
  Growth can be variable depending on the ST, so frequent observation of the culture under a light microscope is recommended.
  Results and validation from this protocol can be found in Valido & Rivera (2007).

## CRYOPRESERVATION

### Basic Protocol 11 Cryopreservation of Xenic *Blastocystis* Cultures

This procedure is based on that of Clark & Stensvold (2016). The procedure should preferably be carried out in a class II biological safety cabinet.

#### Materials

Culture medium base
Appropriate serum (e.g., horse serum or adult bovine serum), heat inactivated
Dimethyl sulfoxide (DMSO)
Xenic *Blastocystis* culture to be cryopreserved

Class II biosafety cabinet
Centrifuge
Cryovials
Isopropyl alcohol freezing container
–80°C freezer or liquid nitrogen (N_2_) freezing container

**Figure 21:**
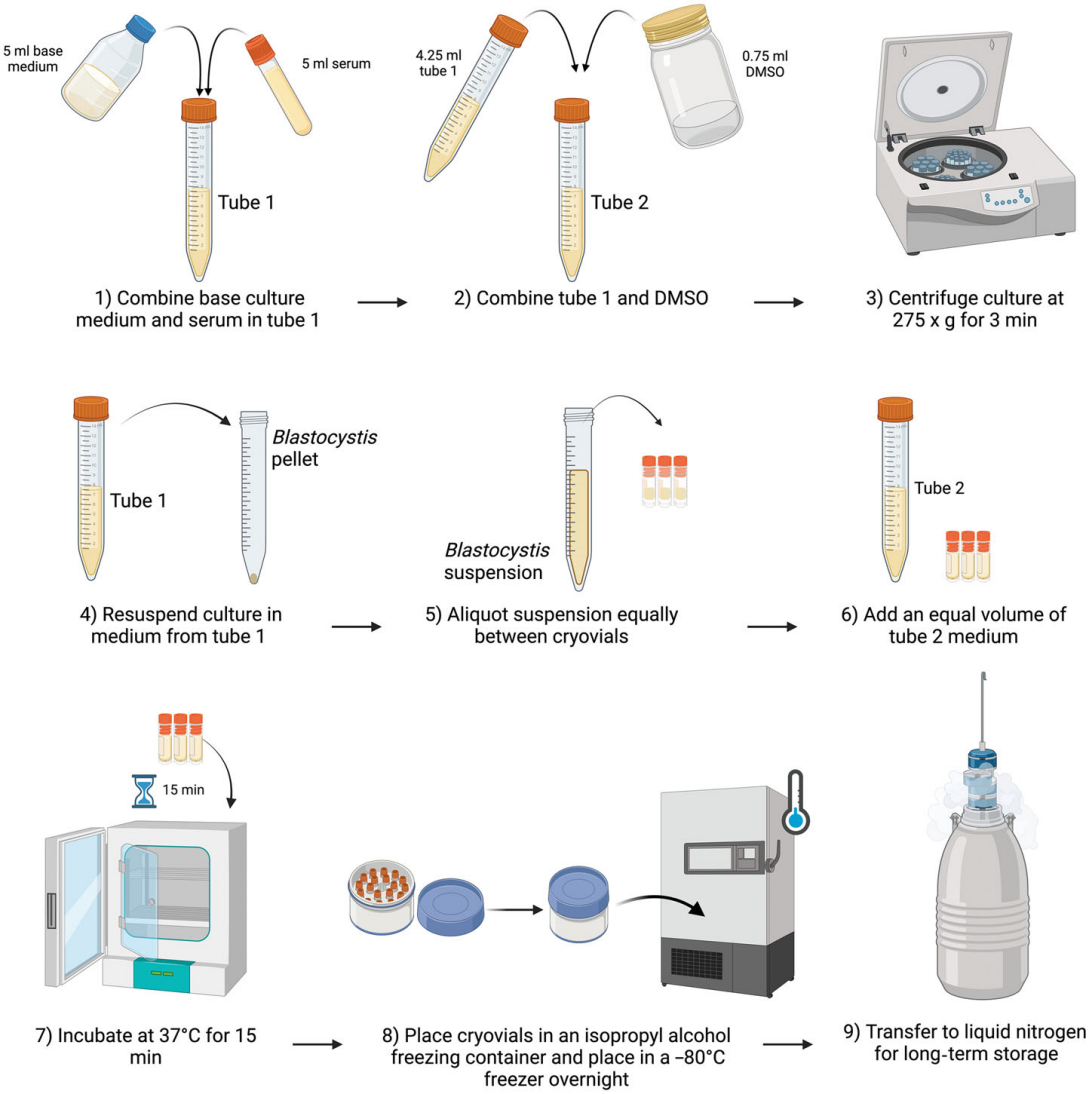
Cryopreservation of xenic cultures. Created in BioRender (D. Shaw, 2025; https://BioRender.com/q61 × 071).

**Figure 22:**
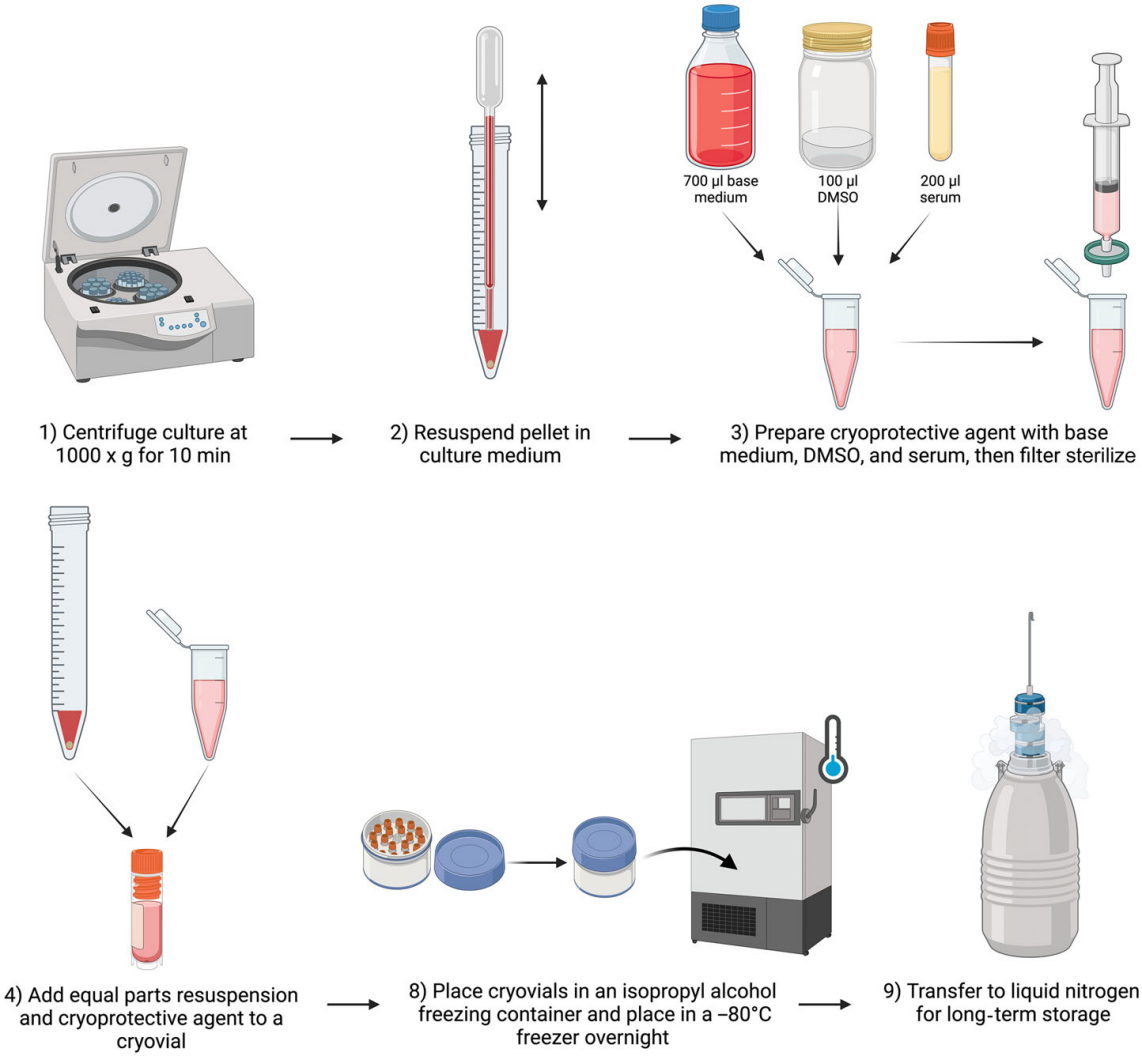
Cryopreservation of axenic cultures. Created in BioRender (D. Shaw, 2025; https://BioRender.com/x16f847).

1 Prepare two tubes:
  a. In tube 1, combine 5 ml culture medium base (without serum) and 5 ml of the appropriate serum.
  b. In tube 2, transfer 4.25 ml from tube 1, add 0.75 ml DMSO, and invert to combine.
2 Working in a class II biosafety cabinet, transfer the culture to be cryopreserved to a centrifuge tube and centrifuge 4 min at 275 × *g*, room temperature
  This pellets the *Blastocystis* cells while leaving most bacterial cells in suspension. As the bacterial cells will survive the cryopreservation process better than *Blastocystis*, this will improve the growth rate of *Blastocysti*s upon revival.
3 Remove and discard the supernatant.
4 Resuspend the *Blastocystis* pellet in medium from tube 1.
5 Aliquot the suspension equally between an appropriate number of cryovials.
  The number of cryovials needed will be dependent on the density of cell growth from the original sample.
6 Add an equal volume of medium from tube 2 and gently mix with a pipet.
7 Incubate at 37°C for 15 min.
  DMSO is used to prevent the formation of ice crystals, which can rupture cells. This incubation allows the DMSO to equilibrate and diffuse into cells.
8 Transfer cryovials to an isopropyl alcohol freezing container and place in a –80°C freezer overnight.
  The isopropanol container reduces the rate of freezing to –1°C/min to reduce cell damage.
9 Once frozen, cultures can be stored at –80°C, but storage in liquid nitrogen (N_2_) is recommended for long‐term storage
  Results and validation from this protocol can be found in Clark & Stensvold (2016); see also Figures 21 and 22.

### Basic Protocol 12 Cryopreservation of Axenic *Blastocystis* Cultures

The following procedure should preferably be carried out in a class II biological safety cabinet.

#### Materials

Axenic *Blastocystis* cultures to be cryopreserved
Liquid IMDM base medium
Appropriate serum (e.g., horse serum or adult bovine serum), heat inactivated
Dimethyl sulfoxide (DMSO)

Class II biosafety cabinet
Centrifuge
0.22‐µm‐pore‐size syringe filter
Cryovials
Isopropyl alcohol freezing container
–80°C freezer or liquid nitrogen (N_2_) freezing container

1 Obtain axenic culture to cryopreserve and, working in a class II biosafety cabinet, transfer to a centrifuge tube.
2 Centrifuge the tube for 10 min at 1000 × *g*, room temperature, to gently pellet the *Blastocystis* cells.
3 Remove as much supernatant as possible without disturbing the pellet.
4 Resuspend in 1 ml IMDM.
5 Prepare 1 ml of cryoprotective agent containing 700 µl IMDM base medium, 200 µl heat‐inactivated horse serum, and 100 µl DMSO.
6 Filter through a 0.22‐µm‐pore‐size syringe filter.
7 Transfer 800 µl of resuspended pellet to a cryovial tube.
8 Add 800 µl cryoprotective agent (from step 5) and mix gently with a pipet.
9 Transfer cryovials to an isopropyl alcohol freezing container and place in a –80°C freezer overnight.
  The isopropanol container reduces the rate of freezing to –1°C/min to reduce cell damage.
10 Once frozen, cultures can be stored at –80°C, but storage in liquid nitrogen (N_2_) is recommended for long‐term storage.

## CULTURING *BLASTOCYSTIS* FROM FROZEN STOCKS

### Basic Protocol 13 Inoculation of Liquid Medium with Xenic *Blastocystis* Cultures from Frozen Stocks

This procedure is based on that of Clark & Stensvold (2016). It should preferably be carried out in a class II biological safety cabinet.

#### Materials

Cryopreserved xenic *Blastocystis* cultures (Basic Protocol 11)
Appropriate culture medium
Class II biosafety cabinet

**Figure 23:**
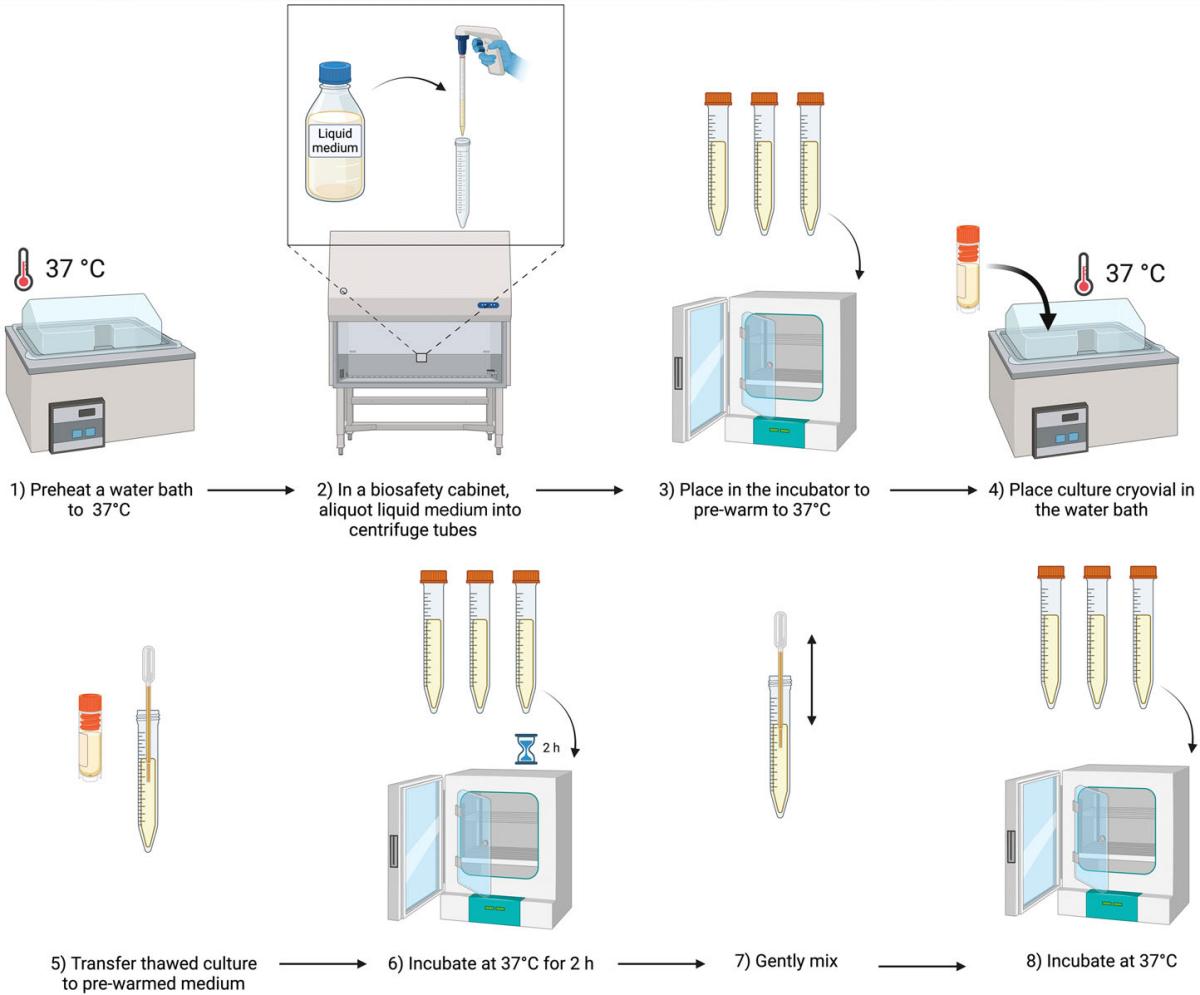
Inoculation of liquid medium with xenic cultures from frozen stocks. Created in BioRender (D. Shaw, 2025); https://BioRender.com/d38v281).

**Figure 24:**
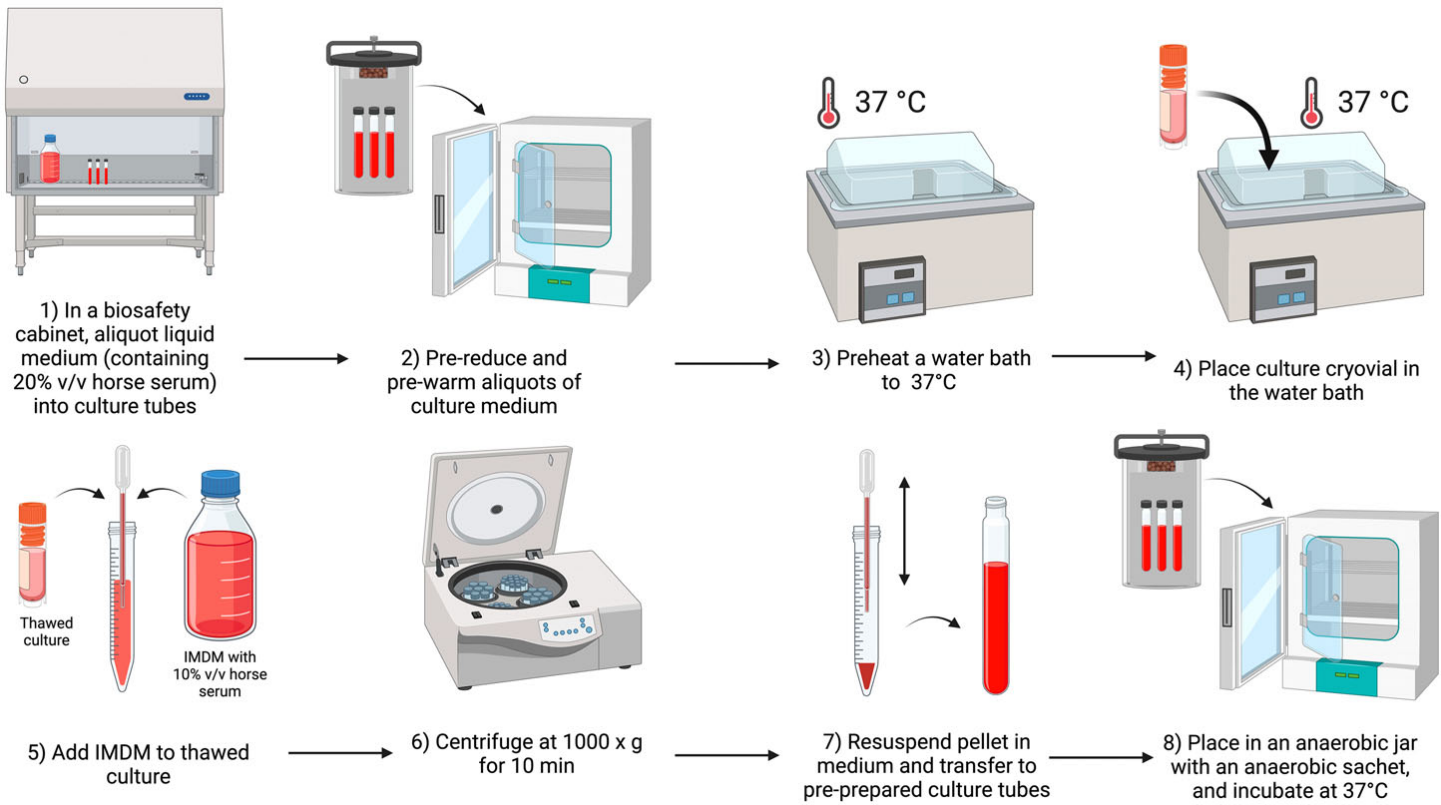
Inoculation of liquid medium with axenic cultures from frozen stocks. Created in BioRender (D. Shaw, 2025; https://BioRender.com/a43y606).

1 Preheat a water bath to 37°C.
2 Pre‐warm aliquots of culture medium to 37°C.
3 Remove a cryovial of culture from liquid nitrogen storage and, working in a class II biosafety cabinet, place it into the water bath, ensuring all contents of the cryovial are immersed.
4 Once thawed, transfer culture to the pre‐warmed medium, incubate for 2 hr at 37°C, and then gently mix by inverting the cryovials a few times by hand.
  DMSO is viscous, so it will settle to the bottom of the culture tube. It is best for the cells to incubate for a while without DMSO being dispersed in the culture medium, before gently mixing it.
5 Incubate at 37°C.
6 Monitor growth over subsequent days and subculture when appropriate.
  Results and validation from this protocol can be found in Clark & Stensvold (2016), as well as Figures 23 and 24.

### Basic Protocol 14 Inoculation of Liquid Medium with Axenic *Blastocystis* Cultures from Frozen Stocks

The following procedure should preferably be carried out in a class II biological safety cabinet.

#### Materials

Cryopreserved axenic *Blastocystis* cultures (Basic Protocol 1^2^)
Appropriate culture medium containing 10% (v/v) heat‐inactivated horse serum

15‐ml centrifuge tube
Class II biosafety cabinet
Centrifuge
Anaerobic jar with anaerobic‐gas‐generating sachets (e.g., Anoxomat III Anaerobic Jar System or the Oxoid AnaeroJar with BD GasPak EZ sachets)

1 Preheat a water bath to 37°C.
2 Pre‐reduce and pre‐warm aliquots of an appropriate culture medium at 37°C.
  Medium for initial inoculation of axenic cultures should contain 20% heat‐inactivated horse serum instead of the 10% usually used for subculturing, because the additional growth factors will help to revive the frozen culture (G.C. Ng, personal communication).
  To pre‐reduce medium, place in anaerobic conditions for 24 hr before use (see below).
3 Remove a cryovial from liquid nitrogen storage and, working in a class II biosafety cabinet, place in the water bath, ensuring that all contents of the cryovial are immersed.
4 Once thawed, transfer the contents of the cryovial to a 15‐ml centrifuge tube and slowly add 10 ml of medium with 10% heat‐inactivated horse serum.
5 Centrifuge the tube for 10 min at 1000 × *g*, room temperature, to gently pellet the *Blastocystis* cells.
6 Remove as much supernatant as possible without disturbing the pellet.
7 Resuspend the pellet in 1 ml of the pre‐reduced, pre‐warmed medium, and then transfer the suspension to the pre‐reduced, pre‐warmed culture medium.
8 Incubate at 37°C anaerobically and subculture every 3‐4 days.
  For anaerobic culture, we recommend using an anaerobic jar system, such as the Anoxomat III Anaerobic Jar System or the Oxoid AnaeroJar with BD GasPak EZ sachets.

## COMMENTARY

### Background Information

The protocols presented in this article represent a significant step toward gathering together all *Blastocystis* spp. culturing techniques and, thus, addressing one of the most challenging aspects of studying this enigmatic protozoan. By providing detailed methodologies for xenic and axenic cultivation, cryopreservation, and the preparation of various media, these tools aim to facilitate reproducibility and enhance the accessibility of *Blastocystis* spp. research across laboratories globally. These protocols also pave the way for large‐scale studies of *Blastocystis* spp. prevalence, ST distribution, and host‐microbe interactions, which are crucial for elucidating its role in health and disease.

#### Impact of current culturing protocols

The ability to culture *Blastocystis* spp. effectively has direct implications for multiple areas of research (Figueiredo et al., 2025). For microbiome studies, xenic cultures provide an opportunity to investigate interactions between *Blastocystis* spp. and other gut microorganisms, shedding light on its ecological role, which is particularly advantageous for epidemiological studies, where obtaining current circulating STs is important. The establishment of culturing media has allowed the extraction of numerous STs from biological STs, which can be used for potential future use in culturing (Lhotská et al., 2020). Although more technically demanding, axenic cultures are invaluable for characterizing this microbe at the molecular and cellular levels without interference from other microbial species. This is particularly critical for genomic, transcriptomic, proteomic, and metabolomic studies, which rely on pure cultures to generate high‐quality, ST‐specific data (Cao et al., 2024). By streamlining xenic cultivation and offering guidance for transitioning to axenic conditions, these protocols contribute to a deeper understanding of *Blastocystis* spp. biology and its impact on the host. In addition, it is essential to highlight the importance of long‐term preservation of cultures, for example in liquid nitrogen, to allow subsequent culturing at future times.

#### Need for more robust axenic culturing protocols

Despite these advances, the field faces significant gaps in the availability of protocols for axenic cultivation, particularly for STs beyond those already successfully agenized (Tsaousis et al., 2024). Multi‐omics approaches, including single‐cell transcriptomics and metabolomics, are increasingly recognized as essential tools for deciphering the complex biology of *Blastocystis* spp. and its interactions with the host and microbiome (Aykur et al., 2024). However, these techniques require consistent access to axenic cultures of diverse STs, which remains a significant bottleneck. Developing more robust, scalable, and ST‐inclusive axenization protocols is therefore a priority for future research.

#### Future directions in blastocystis culturing

Integrating state‐of‐the‐art technologies into *Blastocystis* spp. culturing as the field progresses represents an exciting frontier. Microfluidic platforms, such as gut‐on‐a‐chip technology, offer the potential to mimic the gut environment more precisely, providing controlled conditions for observing *Blastocystis* spp. behavior and growth dynamics in real‐time. Such systems can facilitate high‐throughput culturing and enable studies of environmental factors, such as oxygen gradients and nutrient availability, that influence *Blastocystis* spp. viability. Microfluidic devices may also aid in maximization efforts by isolating individual *Blastocystis* spp. cells and allowing for their growth in defined, sterile microenvironments.

Other innovative methods, such as organoid cultures or co‐culture systems with human intestinal epithelial cells, may provide further insights into *Blastocystis*‐host interactions, including identifying potential immune responses (Deng et al., 2021). These advanced approaches can simulate the host environment, enabling the study of host‐specific responses to *Blastocystis* spp. colonization and elucidating its potential pathogenicity.

Although the protocols presented here provide a strong foundation for *Blastocystis* spp. culturing, further advancements are needed to unlock the potential of this research field fully (Tsaousis et al., 2024). Developing more reliable axenization methods and adopting cutting‐edge technologies will be critical for addressing the current limitations and expanding the scope of *Blastocystis* spp. research. By continuing to refine and innovate these techniques, we can move closer to understanding the multifaceted roles of *Blastocystis* spp. in human and animal health, with implications for diagnostics, therapeutics, and public health strategies.

### Critical Parameters

Although important for the study of *Blastocystis*, its culturing *in vitro* remains a challenge. The main obstacles involve the difficulty of keeping axenic cultures alive, and it is important to remember that different STs have different culturing requirements. Cautions to be aware of include the need to consciously limit the introduction of oxygen into samples to promote optimal growth and survival of the cultures. It is also important to be aware that the protocols detailed here are primarily focused on *Blastocystis* derived from human sources; when working with animal‐derived samples and STs of *Blastocystis*, adjustments to requirements, such as incubation temperature, may be necessary to reflect the host animal's physiology. It is recommended that all culturing of *Blastocystis* be undertaken in a category II biological safety cabinet to prevent contamination, and to prevent thermal shock, medium should always be pre‐warmed to the incubation temperature prior to culturing or subculturing, and pre‐reduced in the case of medium for axenic cultures.

### Troubleshooting

A list of common problems, along with their likely causes and solutions, is provided in Table 2.

**Table 2:** Common Problems That Can Occur When Culturing *Blastocystis*

Problem	Reason	Possible solution
IMDM cultures turning yellow	Most likely bacterial contamination, but may also be due to *Blastocystis* overgrowth	For bacterial contamination, discard the contaminated culture if there are spare stocks or cultures available. If not, treat with 1% (v/v) 10,000 U/ml penicillin/10 mg/ml streptomycin for at least two subcultures.
Axenic cultures not growing well in IMDM	Horse serum can differ depending on origin and supplier	Adjust the pH of complete IMDM to ∼7.6 before filtering can help.
Axenic cultures not growing well in IMDM	IMDM composition can differ depending on supplier	Supplement any missing components of IMDM powder as described in Basic Protocol 6.
Axenic cultures not growing well in IMDM	Possible *Mycoplasma* contamination	Eliminate the *Mycoplasma* infection using an elimination kit, or discard the contaminated culture if there are spare stocks or cultures available.
Cultures in glass tubes are not growing well	Remnants of soap inhibit the cultures	Glass tubes should not be washed with soap; instead, wash with 0.5% HCl, rinse with water, and autoclave.
Cultures in egg media are not growing well	Egg media are porous and may retain gas	Degas the egg solution thoroughly before aliquoting using gentle vacuum or an anaerobic chamber, and dispense the solution slowly to minimize bubble formation.
TYSGM‐9 not filtering	Gastric mucin can clog vacuum filters	First use a filter with a larger pore size to remove any particles not in solution from the mixture of dipotassium phosphate, monopotassium phosphate, sodium chloride, yeast extract, and gastric mucin. Then, add the serum and Tween‐80, and filter using a 0.22‐µm‐pore‐size filter.
Liquid xenic cultures of *Blastocystis* are not growing	Too much oxygen is getting into the culture	Fill culture tubes up to the top with liquid medium to reduce air and create a more microaerophilic environment.

### Author Contributions

**Daisy Shaw**: Conceptualization; visualization; writing—original draft; writing—review and editing. **Constance Denoyelle**: Methodology; writing—review and editing. **Kevin Tan**: Methodology; writing—review and editing. **C. Clark**: Methodology; writing—review and editing. **Hisao Yoshikawa**: Methodology; writing—review and editing. **Eric Viscogliosi**: Methodology; writing—review and editing. **Eleni Gentekaki**: Methodology; supervision; writing—review and editing. **Kateřina** Jirků: Methodology; writing—review and editing. **Anastasios Tsaousis**: Conceptualization; funding acquisition; supervision; writing—original draft; writing—review and editing.

### Conflict of Interest

The authors declare no conflict of interest.

## Data Availability

Data sharing not applicable to this article as no datasets were generated or analyzed during the current study.
